# Single-cell RNA sequencing profiling of mouse endothelial cells in response to pulmonary arterial hypertension^[Author-notes cvab296-FM3]^

**DOI:** 10.1093/cvr/cvab296

**Published:** 2021-11-15

**Authors:** Julie Rodor, Shiau Haln Chen, Jessica P Scanlon, João P Monteiro, Axelle Caudrillier, Sweta Sweta, Katherine Ross Stewart, Alena Shmakova, Ross Dobie, Beth E P Henderson, Kevin Stewart, Patrick W F Hadoke, Mark Southwood, Stephen D Moore, Paul D Upton, Nick W Morrell, Ziwen Li, Stephen Y Chan, Adam Handen, Robert Lafyatis, Laura P M H de Rooij, Neil C Henderson, Peter Carmeliet, Ana Mishel Spiroski, Mairi Brittan, Andrew H Baker

**Affiliations:** Centre for Cardiovascular Science, The Queen’s Medical Research Institute, University of Edinburgh, Edinburgh EH16 4TJ, UK; Centre for Cardiovascular Science, The Queen’s Medical Research Institute, University of Edinburgh, Edinburgh EH16 4TJ, UK; Centre for Cardiovascular Science, The Queen’s Medical Research Institute, University of Edinburgh, Edinburgh EH16 4TJ, UK; Centre for Cardiovascular Science, The Queen’s Medical Research Institute, University of Edinburgh, Edinburgh EH16 4TJ, UK; Centre for Cardiovascular Science, The Queen’s Medical Research Institute, University of Edinburgh, Edinburgh EH16 4TJ, UK; Centre for Cardiovascular Science, The Queen’s Medical Research Institute, University of Edinburgh, Edinburgh EH16 4TJ, UK; Centre for Cardiovascular Science, The Queen’s Medical Research Institute, University of Edinburgh, Edinburgh EH16 4TJ, UK; Centre for Cardiovascular Science, The Queen’s Medical Research Institute, University of Edinburgh, Edinburgh EH16 4TJ, UK; Centre for Inflammation Research, The Queen’s Medical Research Institute, University of Edinburgh, Edinburgh EH16 4TJ, UK; Centre for Inflammation Research, The Queen’s Medical Research Institute, University of Edinburgh, Edinburgh EH16 4TJ, UK; Centre for Cardiovascular Science, The Queen’s Medical Research Institute, University of Edinburgh, Edinburgh EH16 4TJ, UK; Centre for Cardiovascular Science, The Queen’s Medical Research Institute, University of Edinburgh, Edinburgh EH16 4TJ, UK; Department of Medicine, School of Clinical Medicine, University of Cambridge, Cambridge, UK; Department of Medicine, School of Clinical Medicine, University of Cambridge, Cambridge, UK; Department of Medicine, School of Clinical Medicine, University of Cambridge, Cambridge, UK; Department of Medicine, School of Clinical Medicine, University of Cambridge, Cambridge, UK; Centre for Cardiovascular Science, The Queen’s Medical Research Institute, University of Edinburgh, Edinburgh EH16 4TJ, UK; Divisions of Cardiology and Rheumatology, Department of Medicine, Center for Pulmonary Vascular Biology and Medicine, Pittsburgh Heart, Lung, Blood Vascular Medicine Institute, University of Pittsburgh School of Medicine and University of Pittsburgh Medical Center, Pittsburgh, PA, USA; Divisions of Cardiology and Rheumatology, Department of Medicine, Center for Pulmonary Vascular Biology and Medicine, Pittsburgh Heart, Lung, Blood Vascular Medicine Institute, University of Pittsburgh School of Medicine and University of Pittsburgh Medical Center, Pittsburgh, PA, USA; Divisions of Cardiology and Rheumatology, Department of Medicine, Center for Pulmonary Vascular Biology and Medicine, Pittsburgh Heart, Lung, Blood Vascular Medicine Institute, University of Pittsburgh School of Medicine and University of Pittsburgh Medical Center, Pittsburgh, PA, USA; Laboratory of Angiogenesis and Vascular Metabolism, Department of Oncology, Center for Cancer Biology, Leuven Cancer Institute (LKI), VIB and KU Leuven, Leuven 3000, Belgium; Centre for Inflammation Research, The Queen’s Medical Research Institute, University of Edinburgh, Edinburgh EH16 4TJ, UK; Laboratory of Angiogenesis and Vascular Metabolism, Department of Oncology, Center for Cancer Biology, Leuven Cancer Institute (LKI), VIB and KU Leuven, Leuven 3000, Belgium; Centre for Cardiovascular Science, The Queen’s Medical Research Institute, University of Edinburgh, Edinburgh EH16 4TJ, UK; Centre for Cardiovascular Science, The Queen’s Medical Research Institute, University of Edinburgh, Edinburgh EH16 4TJ, UK; Centre for Cardiovascular Science, The Queen’s Medical Research Institute, University of Edinburgh, Edinburgh EH16 4TJ, UK

**Keywords:** Single-cell RNA-seq, PAH, Endothelial cells, Pulmonary hypertension

## Abstract

**Aims:**

Endothelial cell (EC) dysfunction drives the initiation and pathogenesis of pulmonary arterial hypertension (PAH). We aimed to characterize EC dynamics in PAH at single-cell resolution.

**Methods and results:**

We carried out single-cell RNA sequencing (scRNA-seq) of lung ECs isolated from an EC lineage-tracing mouse model in Control and SU5416/hypoxia-induced PAH conditions. EC populations corresponding to distinct lung vessel types, including two discrete capillary populations, were identified in both Control and PAH mice. Differential gene expression analysis revealed global PAH-induced EC changes that were confirmed by bulk RNA-seq. This included upregulation of the major histocompatibility complex class II pathway, supporting a role for ECs in the inflammatory response in PAH. We also identified a PAH response specific to the second capillary EC population including upregulation of genes involved in cell death, cell motility, and angiogenesis. Interestingly, four genes with genetic variants associated with PAH were dysregulated in mouse ECs in PAH. To compare relevance across PAH models and species, we performed a detailed analysis of EC heterogeneity and response to PAH in rats and humans through whole-lung PAH scRNA-seq datasets, revealing that 51% of up-regulated mouse genes were also up-regulated in rat or human PAH. We identified promising new candidates to target endothelial dysfunction including CD74, the knockdown of which regulates EC proliferation and barrier integrity *in vitro*. Finally, with an *in silico* cell ordering approach, we identified zonation-dependent changes across the arteriovenous axis in mouse PAH and showed upregulation of the Serine/threonine-protein kinase Sgk1 at the junction between the macro- and microvasculature.

**Conclusion:**

This study uncovers PAH-induced EC transcriptomic changes at a high resolution, revealing novel targets for potential therapeutic candidate development.

## Graphical Abstract

**Graphical Abstract:**
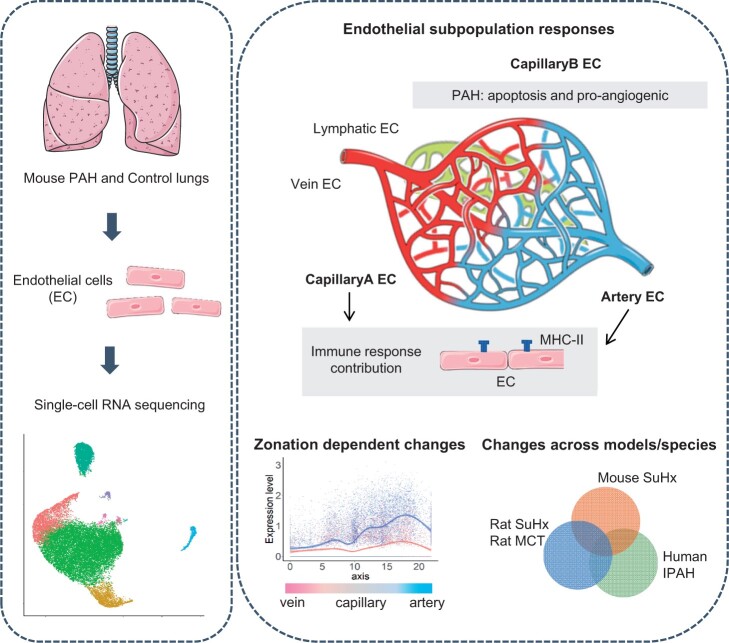

## Introduction

Pulmonary arterial hypertension (PAH) is a rare (15–50 cases per million^1^) but progressive disease characterized by elevated pulmonary arterial pressure (mean > 25 mmHg) and right ventricular hypertrophy.^2^ While treatments to delay disease progression are available, PAH has a poor prognosis with eventual right heart failure and death.^2^ Clinical subtypes include heritable PAH, with mutations most commonly found in the bone morphogenetic protein receptor type II (*BMPR2*) gene, and idiopathic PAH (IPAH).^3^ PAH pathogenesis is complex, involving pulmonary vessel remodelling, enhanced vasoconstriction, and inflammation affecting the arteries and microvasculature.^4^ In humans and some mammals, PAH is also characterized by the presence of plexiform lesions in arterial branching points.^4^ Animal models have been developed to study the pathogenesis of PAH. The widely used SuHx mouse model, which utilizes Sugen 5416 (SU5416) injection and chronic hypoxia (10% O_2_), leads to increased right ventricular systolic pressure (RVSP) and right ventricular hypertrophy.^5,^^6^

Endothelial cells (ECs) are involved in the primary vascular changes leading to PAH.^7^ Subsequent changes include smooth muscle hyperplasia and proliferation contributing to intima remodelling and the recruitment of inflammatory cells. Endothelial injury is common in vascular diseases such as atherosclerosis, peripheral disease,^8^ and pulmonary hypertension.^9^ In PAH, EC apoptosis has been observed in the early stages of the disease, while hyperproliferative apoptosis-resistant ECs may directly contribute to vessel remodelling in later stages.^7^ Loss of endothelium barrier integrity, and altered autocrine and paracrine EC signalling in PAH lead to vasoconstrictor and vasodilator imbalance, and impaired recruitment and/or activation of other cell types.^10^ ECs may also contribute to arterial remodelling via endothelial to mesenchymal transition (EndMT), a process by which ECs acquire mesenchymal phenotypes.^11–13^

Transcriptomic changes in PAH have previously been investigated at the whole-organ and tissue level predominantly using microarray, identifying several genes associated with vascular remodelling and inflammation.^14^ However, as different cell types contribute to PAH throughout its development, these global approaches may hinder the identification of novel targets for therapeutic development. Single-cell RNA sequencing (scRNA-seq) has revolutionized the study of complex tissues in biological and pathological conditions.^15^ In cardiovascular applications, scRNA-seq has improved our understanding of EC development and heterogeneity,^16–18^ the characterization of cell zonation,^19^ and the identification of pathological cell populations.^20^ Recently, scRNA-seq was applied to whole-lung tissues from two different rat models of PAH^21^ and IPAH patient lung tissues,^22^ revealing changes in the distinct pulmonary cell populations, including ECs.^21,^^22^ However, the whole-lung approach does not allow for the study of EC heterogeneity at a high resolution.

Here, we utilized an endothelial lineage-tracing mouse to assess pulmonary EC responses to PAH with scRNA-seq. With a well-established mouse model of pulmonary hypertension which induces right ventricular hypertrophy and increased RVSP,^5,^^6^ we elucidate the dynamic EC responses at a subpopulation level and across the arteriovenous axis. In addition, our dataset is available for interrogation at http://www.mouse-pah.mvm.ed.ac.uk.

## 2. Methods

Extended methods can be found in the Supplementary material online, *Methods*.

### 2.1 Mouse cell line and PAH induction

All animal experiments were performed in accordance with the guidelines from Directive 2010/63/EU of the European Parliament on the protection of animals used for scientific purposes and under the auspices of UK Home Office Project and Personal Licenses held within The University of Edinburgh facilities. *Cdh5-CreERT2-TdTomato* mice were generated by breeding *Cdh5-CreERT2* with *ROSA-TdTomato* [B6.Cg-Gt(ROSA)26^Sortm9(CAG-tdTomato)Hze^] (JAX stock #007909^23^). To achieve induction of Cre, female Cdh5-CreERT2-TdTomato mice were gavaged with 400 mg/kg of tamoxifen, followed by a 2 week wash-out period. To induce PAH in *Cdh5-CreERT2-TdTomato* mice and *C57/BL6* mice, female mice were treated with 3 weeks of weekly 20 mg/kg SU5416 injection, while exposed to chronic hypoxia (10% oxygen) as previously described.^24,^^25^ At the end of the procedure, RVSP was measured under terminal anaesthesia (4% isoflurane) and the mice were euthanized by exsanguination.

### 2.2 scRNA-seq sample preparation and analysis

TdTomato+ mouse lung cells were isolated and sorted as previously described.^26^ ScRNA-seq libraries were prepared using the Single Cell 3′ Reagent Kit User Guide v2 (10× Genomics). Libraries were sequenced on NovaSeq S2 at Edinburgh Genomics. Read mapping and generation of the expression matrix were done with CellRanger using a custom annotation containing the transcript sequence of TdTomato. Low-quality cells were removed using Scater.^27^ The data were normalized using batchelor.^28^ Dimensionality reduction, cluster identification on ‘merged’ or ‘integrated’ data, and differential gene expression analysis were performed with Seurat.^29^ SingleR was used for cell annotation.^30^ Kyoto Encyclopedia of Genes and Genomes (KEGG) Pathway and Gene Ontology analysis and visualization were done using ClusterProfiler,^31^ pathview,^32^ and topGO packages. Cell ordering across the arteriovenous axis was obtained with Slingshot.^33^

Raw and processed data are accessible at the Gene Expression Omnibus (scRNA-seq: GSE154959 and bulk RNA-seq: GSE180169). We also provide data exploration through a Shiny (version 1.5.0) web-based application: http://www.mouse-pah.mvm.ed.ac.uk.

## 3. Results

### 3.1 Study design of mouse pulmonary EC single-cell transcriptomes in Control and PAH

**Figure 1:**
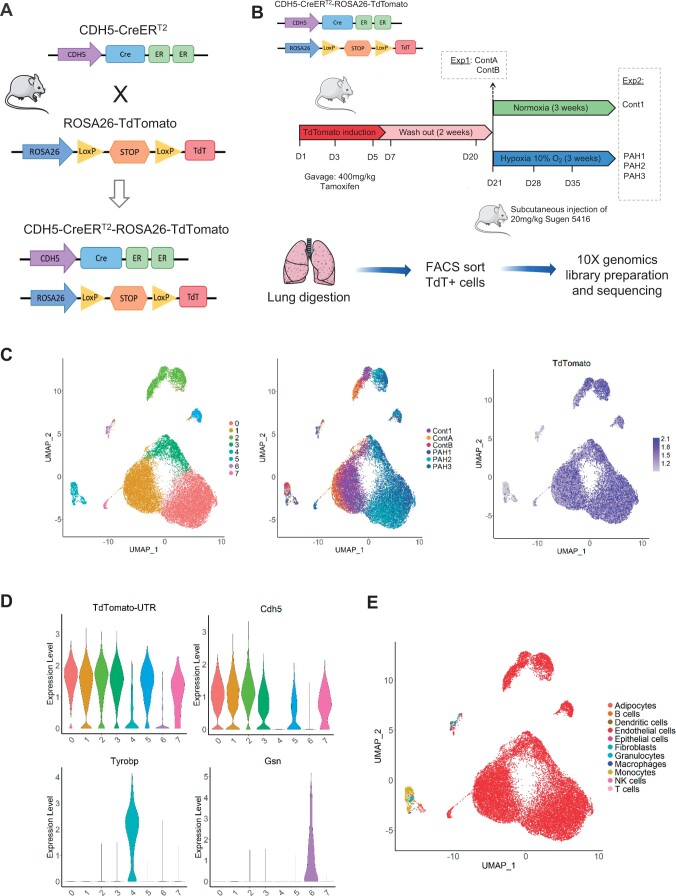
Single-cell RNA-seq of lung ECs in Control and PAH mice. (*A*) Mouse breeding schema to produce the *Cdh5-CreERT2-TdTomato* line. (*B*) Experimental timeline for Experiments 1 and 2. (*C*) Uniform Manifold Approximation and Projection (UMAP) plot of the merged data. Colours represent cell clusters, samples, and TdTomato expression, respectively. (*D*) Violin plot of *TdTomato*, *Cdh5*, *Tyrobp* and *Gsn* expression in the defined clusters. (*E*) UMAP plot of cell identity defined by the tool SingleR.

To study mouse ECs from healthy and PAH lungs, we used a *Cdh5-CreERT2-TdTomato* mouse line (*Figure 1A*), in which the EC-specific Cdh5-driven expression of *TdTomato* is inducible with tamoxifen and maintained in all ECs regardless of subsequent phenotypic changes. After a 2 week tamoxifen wash-out period, TdTomato+ cells from the lungs were isolated using flow cytometry (Supplementary material online, *Figure S1*). We designed two scRNA-seq experiments, allowing the final characterization of Control and PAH TdTomato-positive cells with three replicates per conditions (*Figure 1B*). Experiment 1 aimed to assess the TdTomato+ cell-sorting approach and analyse TdTomato+ cells from two Control lungs, ContA and ContB (*Figure 1B*). Experiment 2 was performed next and included three PAH samples (PAH1, PAH2, PAH3) and one Control (Cont1), kept in normoxic condition (*Figure 1B*). PAH was induced by exposing the *Cdh5-CreERT2-TdTomato* mice to chronic hypoxia for 3 weeks, alongside weekly injections of SU5416. We also performed bulk RNA-seq on TdTomato+ cells from five normoxic mice (bCont1-5) and four SuHx mice (bPAH1-4) to validate our scRNA-seq findings, and collected lung tissues from *C57BL/6* mice in Control and SuHx conditions. We confirmed a significant increase in RVSP and right ventricular hypertrophy in PAH compared with Control mice for both the *Cdh5-CreERT2-TdTomato* and *C57BL/6* lines (Supplementary material online, *Figure S2A*) and a significant increase in the proportion of fully remodelled vessels in PAH *C57BL/6* mice (Supplementary material online, *Figure S2B*).

From Experiment 1, we obtained an average of 3621 cells per mouse with an average 100 273 reads per cell using 10× Genomics scRNA-seq (Supplementary material online, *Figure S3A*). Visualization based on dimensionality reduction using Uniform Manifold Approximation and Projection (UMAP) and clustering revealed the presence of several cellular subpopulations comprising of cells from both mice, showing reproducibility between the two biological samples (Supplementary material online, *Figure S3B*). Three main clusters out of five, corresponding to 88% of the cells, had high *TdTomato* expression, confirming the quality of our TdTomato cell-sorting strategy (Supplementary material online, *Figure S3B*).

For Experiment 2, we obtained between 3162 and 6310 cells per mouse with an average of 127 892 mean reads per cell (Supplementary material online, *Figure S4*).

### 3.2 PAH-induced lung EC transcriptome

Samples from Experiments 1 and 2 were merged, allowing the comparison of three biological replicates per condition (PAH/Cont). UMAP visualization and clustering analysis revealed a clear separation between PAH and Control cells (*Figure 1C*), suggesting a distinct PAH-induced EC transcriptomic profile. These data also showed Control replicates overlapping within the same clusters (*Figure 1C*), despite the independent experimental process and sequencing. *TdTomato* expression analysis confirmed that most clusters (Clusters 0, 1, 2, 3, 5, and 7), which correspond to the majority of cells (95.5%) (Supplementary material online, *Figure S5A*), showed high *TdTomato* expression (*Figure 1C and D*). These clusters also showed high expression of the pan-endothelial markers *Cdh5* (*Figure 1D*) and *Pecam1* (Supplementary material online, *Figure S5B*). There was low *TdTomato* expression in Clusters 4 and 6 (*Figure 1D*), which correspond to only 0.5% of the sequenced cells (Supplementary material online, *Figure S5A*). Marker analysis for these two clusters revealed the presence of immune cell markers and mesenchymal markers, respectively, suggesting that these were non-EC contaminants (*Figure 1D*, Supplementary material online, *Figure S5C*). To confirm the identity of these cells, we use the tool SingleR which infers cell identities using transcriptomic data from pure cell type populations.^30^ As expected, 96% of cells (24 333 out of 25 357) were annotated as ECs while Cluster 4 contained immune cells and Cluster 6 had a high proportion of fibroblasts (*Figure 1E*). This analysis confirmed the high recovery of ECs, with minimal contamination from other cell types, and suggested global maintenance of EC identity in normoxic and PAH-induced conditions.

### 3.3 Limited EndMT in Control and PAH lungs

As EndMT has previously been reported in PAH,^11–13^ we investigated the potential presence of such a population in the scRNA-seq dataset. We could not detect cell populations with high TdTomato expression coupled with low endothelial marker (*Cdh5* and *Pecam1*) expression (*Figure 1C and D*, Supplementary material online, *Figure S6A*) or expression of mesenchymal markers (*Acta2* and *Col1a1*) (Supplementary material online, *Figure S6B*). We also assessed the expression profiles of several EndMT regulators (*Snai1, Snai2*, and *Smad3*), but did not identify cell populations distinctly expressing these markers (Supplementary material online, *Figure S6C*). To further investigate the presence of cells undergoing EndMT, we evaluated the percentage of Acta2+ cells within TdTomato+ cells in the different samples. Less than 1% of TdTomato+ cells expressed Acta2 in both Control and PAH (Supplementary material online, *Figure S6D*). Similar profiles were found when considering Col1a1+ cells (Supplementary material online, *Figure S6E*). We could not confirm the EndMT status of the Acta2+ cells, as they did not show increased expression of the mesenchymal marker *Col1a1* and had comparable EC marker *Cdh5* expression compared with Acta2– cells (Supplementary material online, *Figure S6F*). This suggests that Acta2+ TdTomato+ cells are minimal in the lung and do not seem to be associated with this specific stage of PAH.

### 3.4 Identification of pulmonary ECs subpopulations

**Figure 2:**
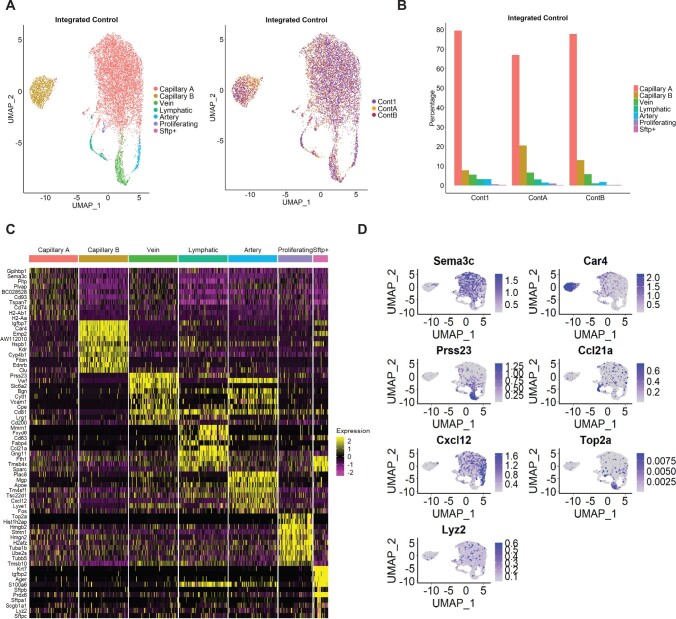
Identification of EC subpopulations in integrated Control samples. (*A*) UMAP plot of integrated Control samples. Colours represent annotated cell clusters and individual sample, respectively. (*B*) Proportion of EC subpopulation in individual Control samples. (*C*) Heatmap of the top 10 marker gene expression in a downsampling of 100 cells from each cluster. (*D*) UMAP plot of representative markers expression in the different clusters.

To further characterize the distinct EC populations in PAH and Control mice, we analysed only cells defined as ‘endothelial cells’ by SingleR. UMAP reduction and clustering of the merged Control samples suggested inter-individual variation, rather than cell type-specific clustering (Supplementary material online, *Figure S7*). Therefore, we used the Seurat integration tool to correct for batch effects, which resulted in seven clusters for the merged Control samples (*Figure 2A*, Supplementary material online, *Table S1*). EC subpopulation identification was based on canonical markers and guided by three recent scRNA-seq of lung ECs.^16,^^34,^^35^ As expected, most ECs (around 70%) belong to a cluster identified as capillary (CapillaryA) (*Figure 2B*), based on *Nrp1* and *Sema3c* enrichment (*Figure 2C and D*). We identified a second capillary cluster, herein defined as CapillaryB, characterized by *Car4* expression, as described previously.^16,^^34,^^35^ Two clusters expressed large vessel markers (*Vwf* and *Vcam1*) and were defined as venous (higher expression of *Vwf* and specific expression of *Prss23*) or arterial ECs (specific expression of *Cxcl12* and *Mgp*) (*Figure 2C and D*). An EC subpopulation with enriched expression of lymphatic EC markers *Ccl21a* and *Prox1* was defined as ‘Lymphatic’. Additionally, we observed a small cluster with high cell cycle-related gene expression, here defined as ‘Proliferating’, and a second small cluster (< 0.4% of cells) defined as ‘*Sftp^+^*’, with high surfactant protein gene (*Sftpa1, Sftpb, Sftpc*, and *Sftpd*) expression (*Figure 2C and D*). Similar analysis of PAH samples detected the same seven clusters (Supplementary material online, *Figure S8*).

**Figure 3:**
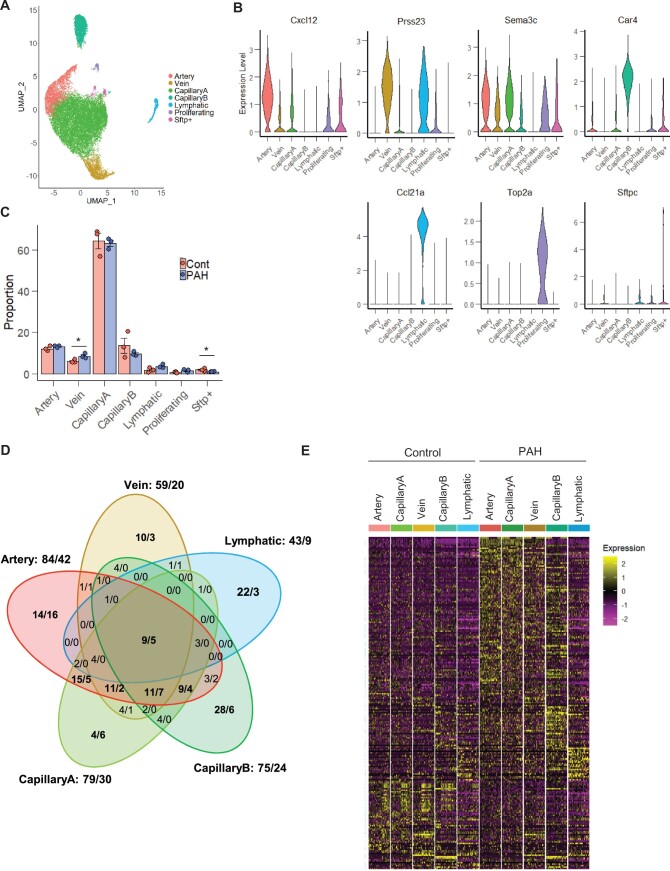
Differential gene expression analysis between PAH and Control in the vessel type EC populations. (*A*) UMAP plot of integrated Control and PAH samples. Colours represent annotated cell clusters. (*B*) Violin plot of vessel type-specific markers expression in the annotated EC subpopulations. (*C*) Proportion of the annotated EC subpopulations in Control and PAH samples. Error bars correspond to standard error of the mean. P-value obtained using an unpaired t-test on the log10 proportion (*P-value <0.05). (*D*) Venn diagram of differential gene expression changes (number of upregulated genes/number of down-regulated genes) in the five vessel type EC subpopulations. (*E*) Heatmap of all differentially expressed genes across vessel type EC subpopulations and conditions in a downsampling of 50 cells per category.

### 3.5 EC subpopulation responses in PAH

To define the transcriptional changes mediated by PAH in EC subpopulations, we integrated all Control and PAH samples. The seven subpopulations identified in the separate analysis of Control and PAH were also identified in this integrated analysis (*Figure 3A and B*). PAH samples showed a slightly higher proportion of Vein ECs compared with Control samples and similar proportion of the four other vessel type-specific ECs (i.e. Artery, CapillaryA, CapillaryB, and Lymphatic ECs) (*Figure 3C*). The relative proportion of proliferative ECs was constant between Control and PAH lungs (*Figure 3C*). As human PAH is often associated with increased EC proliferation,^5,^^7^ we also assessed the percentage of cells in each cell cycle phase in each individual cluster and across all ECs but did not detect any significant differences between PAH and Control (Supplementary material online, *Figure S9*), suggesting that the proportion of Proliferating ECs is not increased at this stage of the SuHx model.

**Figure 4:**
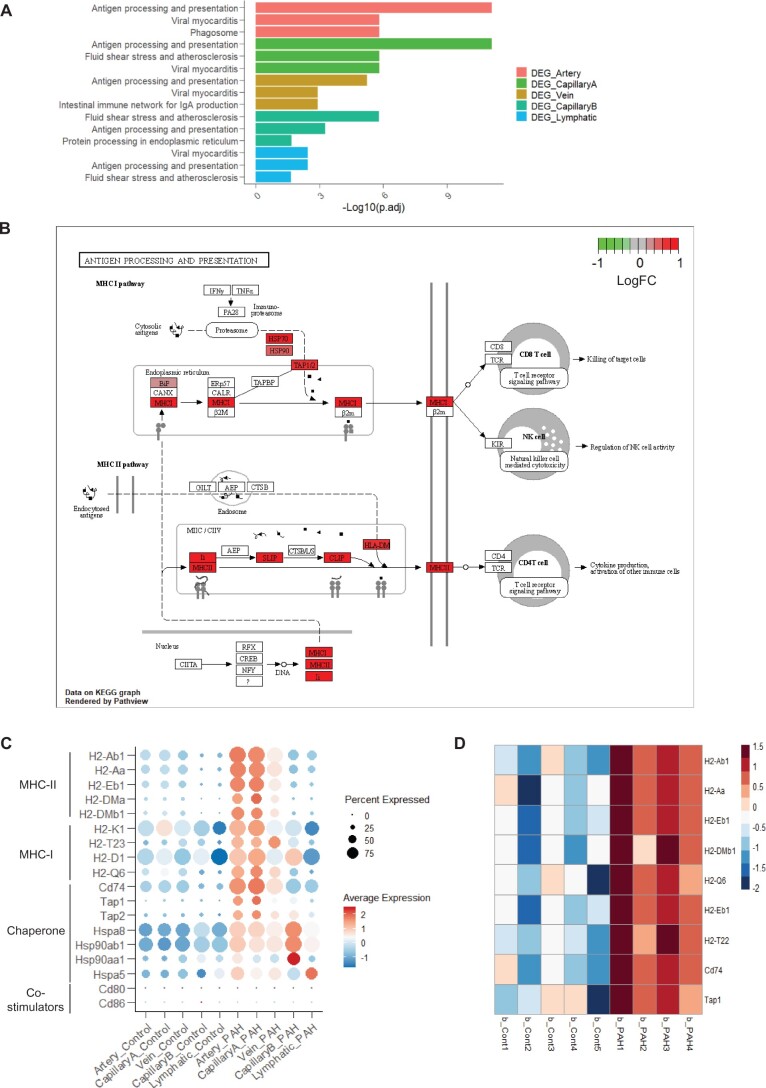
Activation of the antigen processing and presentation pathway in ECs in PAH. (*A*) Top 3 enriched KEGG pathways for each vessel type DEG. (*B*) Visualization of the Artery DEGs on the ‘Antigen Processing and Presentation’ pathway graph. (*C*) Dot plot showing the expression of DEG annotated in the KEGG ‘Antigen Processing and Presentation’ pathway and their co-stimulators across the EC subpopulations and conditions. (*D*) Heatmap [z-score of Log2(FPKM+1)] of significant genes involved in the Antigen Processing and Presentation pathway in the bulk RNA-seq of TdTomato+ cells.

We performed a differential gene expression analysis in each of the vessel type-specific EC clusters to identify PAH-dependent changes. Global and vessel type-specific changes were identified with a total of 222 significant differentially expressed genes (DEGs) detected, based on a log fold change of 0.25 (*Figure 3D*, Supplementary material online, *Table S2*). This analysis revealed a greater number of DEGs in Artery, CapillaryA, and CapillaryB ECs compared with Vein and Lymphatic ECs (*Figure 3D*). Some DEGs were commonly regulated in Artery, CapillaryA, and Vein ECs, while CapillaryB and Lymphatic ECs exhibited subpopulation-specific transcriptomic responses to PAH (*Figure 3D and E*). For each EC subpopulation, we assessed the expression of the DEGs across the three Control and three PAH biological replicates and confirmed comparable responses across all replicates (Supplementary material online, *Figure S10*). We also validated the changes of 42 genes (out of the 222 DEGs) in additional replicates using the bulk RNA-seq dataset (Supplementary material online, *Figure S11A*). PCA analysis of the bulk RNA-seq confirmed the distinct profiles of the Control and PAH samples (Supplementary material online, *Figure S11B*) and differential gene expression analysis identified 345 and 689 significant up- and down-regulated genes, respectively, based on a 1.5-fold change (Supplementary material online, *Table S3*). As bulk RNA-seq averages gene expression, we expect a higher validation of changes detected in the largest cell populations from the scRNA-seq. Delimiting our scRNA-seq analysis to a 1.5-fold change threshold in CapillaryA, 56% of DEGs could be validated in the bulk RNA-seq data (Supplementary material online, *Figure S11A*).

In addition to the vessel type EC clusters, we also analysed DEGs in the Proliferating EC clusters in PAH and Control. From the 42 significantly regulated genes (35 up-regulated, 7 down-regulated), 36 genes were also differentially expressed in the vessel type EC clusters (Supplementary material online, *Figure S12A*), suggesting that Proliferating ECs did not show a PAH-specific transcriptional response.

In the scRNA-seq dataset, we also noticed 10 genes displaying upregulation in PAH1 and PAH3 but not PAH2 (Supplementary material online, *Figure S13A*). Four of these genes were previously reported as downstream targets of the transcription factor, aryl hydrocarbon receptor (AhR),^36,^^37^ which is activated by SU5416,^38^ suggesting that PAH2 had a limited response to SU5416 treatment. However, up-regulation of six genes was validated in the bulk RNA-seq data (Supplementary material online, *Figure S13B*), confirming their relevance to the SuHx model.

To address inter-individual variability and identify high confidence candidates, we performed a stringent analysis of the scRNA-seq dataset. By comparing all individual PAH to all Controls samples and focusing on common changes, we obtained a list of 30 DEGs (Supplementary material online, *Figure S14* and *Table S2*). The lower number of cells in each comparison had less power to identify significant genes, hence the shorter DEG list, but this stringent approach gave priority to candidates with high and consistent changes. We confirmed the dysregulation of 14 genes in the bulk RNA-seq (Supplementary material online, *Table S3*).

### 3.6 PAH-induced activation of the antigen processing and presentation pathway in ECs

To understand the functional effects of these transcriptional changes, we performed a KEGG pathway enrichment analysis with the 222 DEGs identified in the group analysis (*Figure 3D*). The antigen processing and presentation pathway, involved in T-cell recruitment and activation,^39^ was enriched across all vessel type ECs (*Figure 4A*) and Proliferating cells (Supplementary material online, *Figure S12B*). Seventeen genes from distinct segments of this pathway were up-regulated in Artery ECs in PAH (*Figure 4B*). The highest upregulation was observed for the major histocompatibility complex class II (MHC-II) and its chaperone, *Cd74*, in Artery and CapillaryA ECs (*Figure 4C*). However, the *Cd80* and *Cd86* co-stimulatory molecules required for naïve T-cell activation^39^ showed low expression in both Control and PAH (*Figure 4C*). The up-regulation of genes relevant to the antigen processing and presentation pathway was confirmed in the bulk RNA-seq (*Figure 4D*).

**Figure 5:**
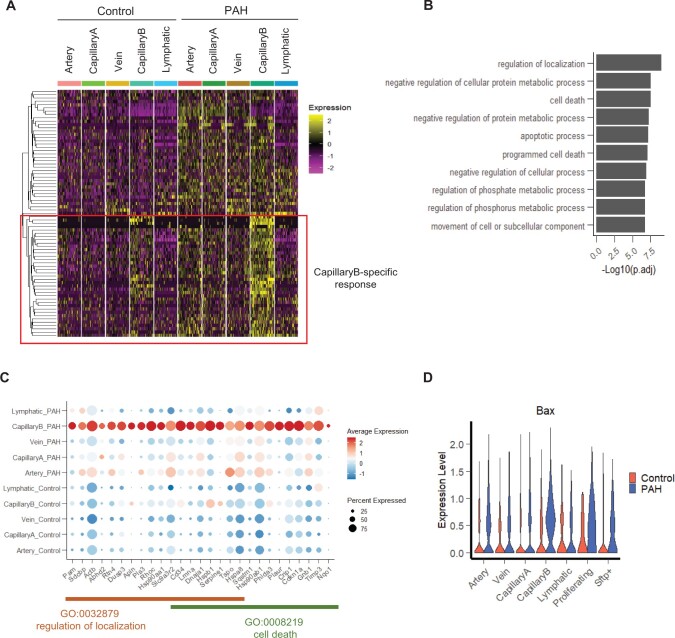
Characterization of the PAH response in CapillaryB EC subpopulation. (*A*) Heatmap of up-regulated genes in CapillaryB in a downsampling of 50 cells per category. A hierarchical clustering approach was used to identify genes with a specific up-regulation in CapillaryB compared to the other EC populations. (*B*) Top 10 enriched Go Terms (Biological Process) of the CapillaryB-specific up-regulated genes. (*C*) Dot plot showing the expression of genes specifically up-regulated in CapillaryB and annotated in the ‘regulation of localization’ and ‘cell death’ Go Terms. (*D*) Violin plot of *Bax* expression across EC populations and conditions.

### 3.7 PAH regulation of apoptosis, pro-migratory, and pro-angiogenic genes in CapillaryB ECs

To identify PAH-mediated gene up-regulation specific to CapillaryB ECs, we performed a hierarchical clustering of all CapillaryB DEGs based on their expression profiles across all EC subpopulations and conditions, and focussed on 37 genes showing a stronger response to PAH in CapillaryB (*Figure 5A*). Gene Ontology analysis revealed that these genes are involved in the regulation of localization and cell death (*Figure 5B and C*). EC cell death has been observed in early-stage PAH, with a peak of apoptotic cells detected at 1 week in the SuHx mouse model, followed by a longitudinal decrease.^5^ Therefore, increased apoptotic cell numbers is not expected in the current study. Additionally, as the cell preparation for scRNA-seq includes a live cell selection, apoptotic cells, specifically late apoptotic cells, might not be represented in the scRNA-seq dataset. We did not observe a difference in the number of cells with high mitochondrial genes (i.e. apoptotic cells) between Control and PAH during scRNA-seq quality control and filtering (Supplementary material online, *Figures S3A and S4*), suggesting apoptotic cells might not be associated with this stage of PAH. However, as changes in apoptotic regulatory genes may still be detectable, we analysed the signature score of genes involved in the execution of apoptosis (based on Gene Ontology GO:0097194) and positive and negative regulation of apoptosis (Go terms GO:0043065 and GO:0043066). While the expression of the execution phase of apoptosis genes was negligible across all EC types and condition (Supplementary material online, *Figure S15A*), we observed an increase in expression of both positive and negative apoptotic regulatory genes in PAH (Supplementary material online, *Figure S15B*), likely reflecting the ongoing regulation of apoptosis following the 1 week peak. We noted the significant up-regulation of the pro-apoptotic regulator Bax in CapillaryB in PAH (*Figure 5D*).

**Figure 6:**
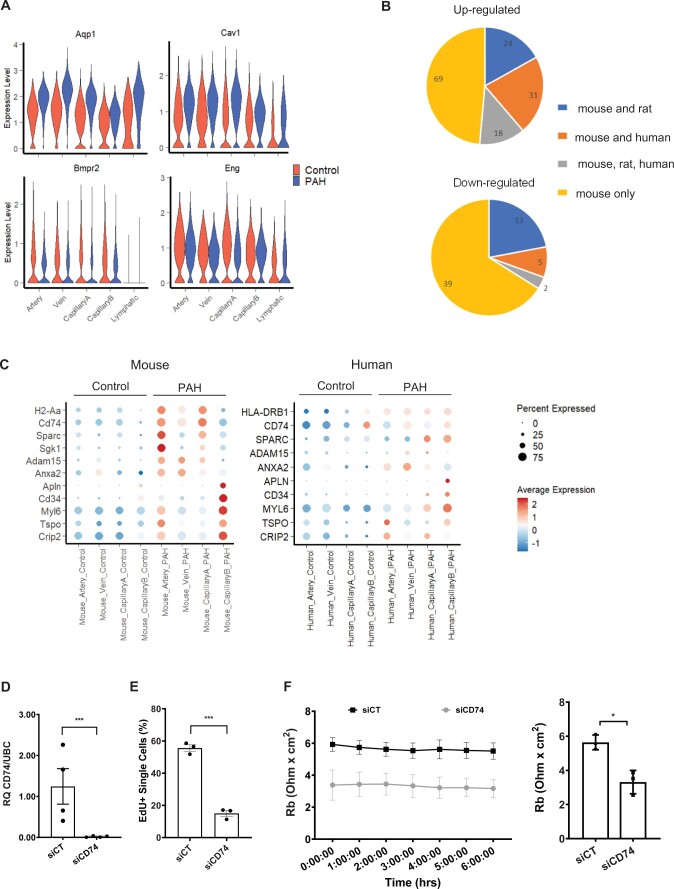
Comparison of the mouse PAH DEGs with human genetics and transcriptomics data. (*A*) Violin plot showing the expression of 4 DEGs with PAH-associated variants, across EC populations and conditions. (*B*) Number of mouse up/down-regulated genes regulated in the same direction in rat or human PAH ECs. (*C*) Dot plot showing the expression of selected candidates across EC populations and conditions in mouse and human scRNA-seq. (*D*) Expression of *CD74* in Control (siCT) and CD74 (siCD74) knockdown HUVECs by RT-qPCR. RQ, relative quantification normalized to *UBC* relative to siCT (*n* = 4). (*E*) Quantification of EdU uptake in siCT and siCD74 HUVECs (*n* = 3). (*F*) Cell-to cell interaction, expressed as Rb (Ohm × cm^2^), in siCT and siCD74 HUVECs across a 6 h time course with bar graph showing the average across the time points (*n* = 3). Graph in panels *D*–*F* correspond to mean ± standard error of the mean and *P*-values were obtained using an unpaired *t*-test. *P-value < 0.05 and ***P-value < 0.0001.

Among the 37 genes with CapillaryB-specific changes, we also noticed the presence of three known tip cell-enriched genes: *Cd34*, Plasminogen Activator Urokinase Receptor (*Plaur*) and Apelin (*Apln*). Tip cells are localized at the tips of growing vessels during sprouting angiogenesis^40,^^41^ and are characterized by the expression of *Dll4, Angpt2, Cxcr4*, and *Apln.*^42,^^43^ We assessed the expression of these markers in the scRNA-seq but a tip cell subpopulation could not be identified (Supplementary material online, *Figure S16A*) and only *Apln* was enriched in CapillaryB ECs (*Figure 5C*, Supplementary material online, *Figure S16A*). In agreement with a lack of tip cells, the expression of genes involved in sprouting angiogenesis (GO:0002040) was negligible across EC subpopulations and conditions (Supplementary material online, *Figure S16B*). In contrast, we observed a higher gene expression for positive, but not negative, regulators of angiogenesis (GO:0045766 and GO:0016525) in CapillaryB ECs, with the PAH group having a higher expression than Control (Supplementary material online, *Figure S16C*). These data suggest angiogenic regulatory pathways are activated in CapillaryB ECs and enhanced in PAH. Interestingly, 10 out of the 37 CapillaryB-specific DEGs are also among the top 50 markers of CapillaryB ECs in Control (Supplementary material online, *Table S1*), suggesting that characteristics of CapillaryB ECs were enhanced in response to PAH.

Overall, we showed PAH-mediated regulation of apoptotic, pro-migratory, and pro-angiogenic genes in CapillaryB ECs.

### 3.8 Relevance of PAH-mediated mouse EC changes in rat and human PAH

To evaluate the relevance of the SuHx mouse scRNA-seq data in human PAH, we examined whether the expression of human genes with PAH-associated variants were also altered in mouse PAH ECs. From the 12 high-confidence genetic drivers of PAH,^3^ four genes were identified: Aquaporin (*Aqp1*), Caveolin1 (*Cav1*), *Bmpr2*, and Endoglin (*Eng*). *Aqp1*, with the highest fold change and part of the stringent DEG set, was up-regulated in Artery, Vein, CapillaryA, and Lymphatic ECs (*Figure 6A*). *Cav1* was also up-regulated while *Bmpr2* and *Eng* were down-regulated (*Figure 6A*).

**Figure 7:**
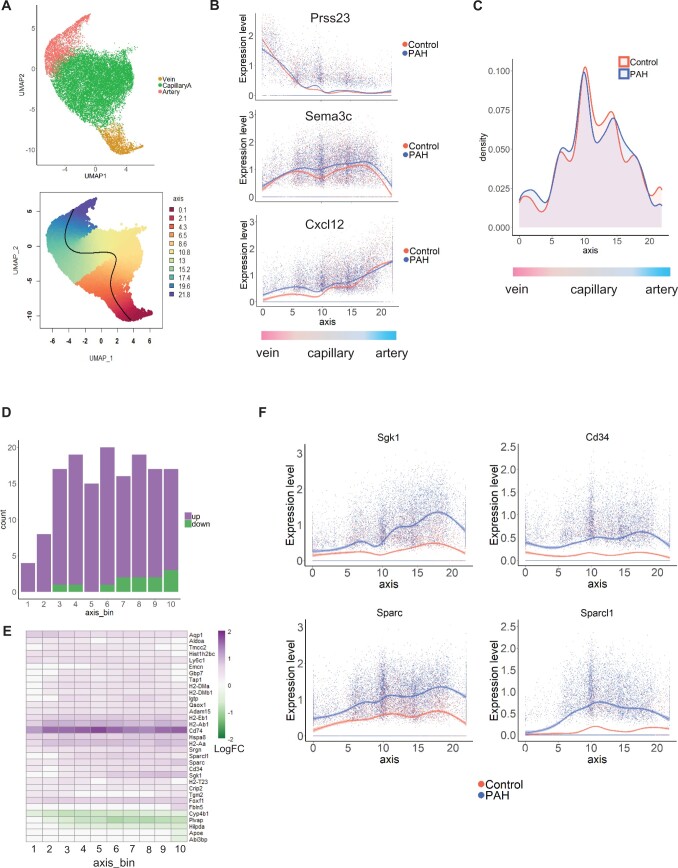
Differential gene expression changes across the arteriovenous axis. (*A*) UMAP plot of Artery, CapillaryA and Vein selected clusters. Colours correspond to EC subpopulations and trajectory unit, respectively. Trajectory arbitrary unit corresponding to the arteriovenous axis unit and trajectory line were obtained with Slingshot. (*B*) Expression of the vein marker *Prss23*, capillary marker *Sema3c* and artery marker *Cxcl12* in Control and PAH cells ordered along the arteriovenous axis. (*C*) Cell density across the arteriovenous axis in Control and PAH groups. (*D*) Differential gene expression changes in 10 distinct sections of the arteriovenous axis based on a stringent analysis of individual samples. (*E*) Heatmap of the stringent DEG Log Fold change across 10 distinct sections of the arteriovenous axis. (*F*) Expression profile across the arteriovenous axis in Control and PAH conditions for *Sgk1, Sparc, Sparcl1*, and *Cd34*.

We also mined recent rat^21^ and human PAH^22^ whole-lung scRNA-seq datasets. The rat dataset includes two different models of PAH: SuHx and monocrotaline (MCT). We retrieved data corresponding to the 758 annotated ECs, ranging from 1 to 343 per sample (Supplementary material online, *Figure S17A*). Due to the low number of rat ECs, we integrated the rat with the mouse EC dataset, and obtained seven EC subpopulations, per the mouse analysis (Supplementary material online, *Figure S17B–D*). Rat Control, SuHx, and MCT ECs were present in all seven clusters (Supplementary material online, *Figure S17D*) and expressed similar EC subpopulation markers as the mouse ECs (Supplementary material online, *Figure S17E*). The human dataset, which included six Controls and three IPAH samples, was analysed similarly to the mouse dataset starting from the raw sequencing data. After dimensional reduction and clustering, Cluster 3 was annotated as ECs based on the enriched expression of several EC markers including *CDH5* (Supplementary material online, *Figure S18A–C*). We identified 3950 ECs (45–1137 per sample) across Control and IPAH (Supplementary material online, *Figure S18D*). To identify EC subpopulations, we selected all ECs and performed a new dimensional reduction and clustering analysis after sample integration to take sample variation into account. We obtained seven clusters, five of which correspond to different vessel types (Artery, Vein, CapillaryA, CapillaryB, and Lymphatic), and also identified bronchial ECs, as previously described in lung scRNA-seq,^44^ and three minor clusters, not annotated in this study.

To compare PAH-induced EC response across species, we performed a differential expression analysis between PAH and Control in the rat and human data for the four blood vessel type EC subpopulations (Artery, Vein, CapillaryA, and CapillaryB). We obtained 991 DEGs in human ECs using the same threshold per the mouse analysis (Supplementary material online, *Table S4*). We identified 884 DEGs in rat ECs with a similar analysis but without multiple comparison corrections as the number of ECs was low in the different clusters and conditions (Supplementary material online, *Table S5*). Overall, we found that 51% of the up-regulated mouse genes (14% of the mouse down-regulated genes) were also differentially expressed in rat or human, and found 20 genes commonly regulated across all three species (*Figure 6B*). As Artery and CapillaryA ECs have a high number of up-regulated genes in mouse PAH, we analysed the DEG overlap in these two EC subpopulations (Supplementary material online, *Figure S19*). Interestingly, three (*Cd74, Sparc, Slc6a6*) and five (*Sparc, Cd81, Anxa2, Id3, Slc9a3r2*) genes were up-regulated in mouse, rat, and human Artery and CapillaryA ECs, respectively (Supplementary material online, *Figure S19*). In addition to *CD74*, genes in the MHC-II complex (HLA genes) were also up-regulated in human IPAH, suggesting the importance of this pathway (*Figure 6C*, Supplementary material online, *Figure S19*). Several genes, including *Adam15* and Serum/Glucocorticoid Regulated Kinase 1 (*Sgk1*), were up-regulated in all species but with some differences in the expressing EC subpopulation (*Figure 6C*, Supplementary material online, *Figure S19*), likely reflecting species-specific regulation or variability in categorizing artery, vein, and capillary EC clusters within the arteriovascular network. We observed SuHx-specific up-regulation of *Cyp1a1* and *Cyp1b1* in rat ECs (Supplementary material online, *Figure S17F*), in agreement with a regulation by SU5416. Finally, five genes showing CapillaryB specificity in mouse were also up-regulated in human CapillaryB ECs in PAH, with *APLN, CD31* and *MYL6* specifically enriched in this subpopulation in human (*Figure 6C*).

To determine functional relevance of the identified targets, we selected *CD74* for its global alteration across mouse vessel type ECs in PAH and its regulation in rat and human datasets. Increased CD74 protein levels in IPAH ECs were previously reported from immunostaining of human IPAH tissues and western blot of isolated IPAH ECs.^45^ Additionally, CD74 contributed to the recruitment of peripheral blood mononuclear cells to pulmonary ECs *in vitro*,^45^ supporting the involvement of the CD74/MHC-II complex in PAH. As *CD74* also affects cell proliferation in other cell types, including epithelial cells,^46^ we aimed to further characterize the role of *CD74* via gene knockdown in human umbilical vein endothelial cells (*Figure 6D*). *CD74* depletion led to a decrease in EC proliferation measured by 5-ethynyl-2’-deoxyuridine (EdU) incorporation (*Figure 6E*, Supplementary material online, *Figure S20A*), and a loss of barrier resistance (Supplementary material online, *Figure S20B*), specifically cell–cell interaction (*Figure 6F*) but not cell–matrix interaction (Supplementary material online, *Figure S20C*). These data support the important contribution of *CD74* to EC function.

### 3.9 Mapping transcriptomic changes across the arteriovenous axis in PAH

From the UMAP visualization shown in *Figure 3A*, we observed Artery and Vein EC clusters attached to either side of the CapillaryA EC cluster, recapitulating the continuous lung vascular architecture. To study EC zonation across the arteriovenous axis, we performed an *in silico* lineage-tracing analysis using Slingshot.^33^ Cells were ordered along the arteriovenous axis (*Figure 7A*) and the expression of Vein, CapillaryA, and Artery markers, *Prss23, Sema3c*, and *Cxcl12*, respectively, were used to confirm a gradient of expression along the vasculature (*Figure 7B*). Control and PAH ECs were found across the arteriovenous axis, with slight differences in their distribution (*Figure 7C*). PAH cells were less distributed in large arteries and in the arterial side of the microvasculature (*Figure 7C*). This observation might reflect the enlarged arterioles and loss of distal vessels which are characteristic of PAH.^4^ We carried out a stringent differential gene expression analysis in 10 sections along this axis, identifying 33 DEGs, with a lower number of DEGs in the venous region of the axis (*Figure 7D*). This analysis revealed zonation-dependent changes (*Figure 7E*) with the *Sgk1* and *Cd34* genes displaying a peak of upregulation at the junction between capillary and arterial ECs (*Figure 7E and F*). Two genes from the secreted protein acidic and rich in cysteine (SPARC) family of proteins also showed different expression profiles, with *Sparc* up-regulated in ECs from arteries and *Sparcl1* up-regulated in the microvasculature (*Figure 7E and F*).

## 4. Discussion

To characterize PAH-induced EC molecular changes at the single-cell level, we performed scRNA-seq analysis across three SuHx-mediated PAH and three Control mice. Sorted EC sequencing enabled high resolution identification of PAH-induced EC responses at a subpopulation level and across the arteriovenous zonation. We showed the strong activation of the MHC-II pathway in Artery and CapillaryA ECs and the specific upregulation of pro-migratory and pro-angiogenic genes in CapillaryB ECs in PAH. By comparing with rat and human genetic and transcriptomic data, we demonstrated the relevance of this mouse data across models and to human disease. We also identified promising and novel candidates regulated in ECs in PAH, specifically *CD74*, which is involved in the regulation of EC proliferation and barrier function *in vitro*. We also developed a web-based application for interactive exploration of this scRNA-seq data (http://www.mouse-pah.mvm.ed.ac.uk).

Using the *Cdh5-CreERT2-TdTomato* mouse line, we identified five main EC clusters corresponding to the different lung vessel types (Artery, Vein, CapillaryA, CapillaryB, and Lymphatic), previously identified with different EC isolation strategies based on the surface markers CD31^16,^^34^ or ICAM2.^35^ Interestingly, our analysis of publicly available rat^21^ and human^22^ PAH scRNA-seq datasets also revealed the presence of these five distinct subpopulations in rat and human PAH lung tissues. In mouse, we also identified two additional small clusters annotated as ‘Proliferating’ and ‘Sftp^+^’. Proliferating ECs are sometimes found in healthy tissues at a low level,^16,^^35^ while ‘Sftp^+^’ cluster corresponded to cells expressing high level of surfactant protein genes. As *Sftp* genes are highly expressed in alveolar type 2 cells (AT2),^47^ further work is required to determine if these cells are AT2 contaminants or a novel EC subtype.

No major changes in cell population proportions were observed between Control and PAH mice. We noted a slight increase in the relative proportion of vein ECs in PAH. While this could indicate an absolute increase of vein ECs, this change could also reflect pruning of the distal vasculature leading to a change in the relative proportion of vein ECs if other vessel types regressed. Rat and human EC analysis also showed similar EC population proportions between PAH and Control samples, suggesting persistence of cell type identity and relative numbers, but with associated transcriptional changes. While late-stage PAH has previously been associated with EC proliferation,^7^ the scRNA-seq suggest that EC proliferation is not evident at this time point in the SuHx model of PAH.

The use of the mouse *Cdh5-CreERT2-TdTomato* line allowed us to assess the contribution of EndMT in Control and PAH lungs. We did not identify any cell populations with high TdTomato level and high expression of EndMT markers and/or regulators. In our initial clustering, two small clusters showed non-EC marker expression. However, these two clusters also showed a low level of TdTomato expression, suggesting the presence of contaminants rather than transitioned ECs. The low proportion of TdTomato+/Acta2+ cells in both Control and PAH samples also suggests a minimal contribution of EndMT at this stage of PAH. Previous studies have shown the presence of EndMT in the SuHx mouse model using immunofluorescence and flow cytometry.^13^ These differences could be explained by the sensitivity limitations of 10× Genomics scRNA-seq technology for low expressed genes and/or the transient and reversible nature of EndMT, which has been confirmed in recent scRNA-seq of ECs after myocardial infarction.^48^ Further studies combining diverse detection methods and different pathological models across time points are required to confirm the contribution of EndMT in PAH at different stages of the disease.

Our joint analysis of Control and PAH, combining three animals per group, revealed 222 DEGs across the five vessel type EC clusters. Overall, we found high reproducibility across replicates even when integrating two independent experiments, and confirmed the regulation of many candidates in additional mouse samples using bulk RNA-seq. DEGs showing inter-individual differences in PAH mouse scRNA-seq included four direct targets of the transcription factor AhR^36,^^37^ such as *Cyp1a1* and *Cyp1b1*, up-regulated in PAH1 and PAH3 but not PAH2. In the rat SuHx model, SU5416 may exacerbate PAH through the activation of AhR,^38^ suggesting that PAH2 had a reduced response to SU5416 treatment. All three PAH mice showed comparable RVSP and right ventricular hypertrophy, indicating that the AhR pathway is not necessary to induce PAH but may contribute to PAH progression. In agreement, *Cyp1a1* and *Cyp1b1* were found up-regulated in the rat SuHx scRNA-seq data but not in the MCT model nor in the human IPAH samples. Further work, including a larger mouse cohort and more in-depth phenotypic characterization, is required to dissect the contribution of SU5416 vs. hypoxia in PAH phenotypes.

The largest change in PAH was the upregulation of MHC-II genes, affecting all ECs and particularly Artery and CapillaryA ECs (*Figure 4*). MHC-II genes are expressed by professional antigen-presenting cells^39^ and ECs under inflammatory conditions.^49^ Our data suggests that this activation occurs in PAH. We did not detect the up-regulation of MHC-II co-stimulatory molecules such as *Cd80* and *Cd86*, suggesting that in PAH, ECs can contribute to the activation of antigen-experienced T-cells,^50^ or to T-cell adhesion,^51^ but not to the activation of naïve T-cells. In human studies, single-nucleotide polymorphisms and allele frequency of the MHC-II genes, *HLA-DPA1* and *HLA-DPB1*, have been associated with PAH.^52^ The effects of these variants on the pulmonary vasculature warrant further investigation.

In contrast to the pan EC DEGs, we identified a CapillaryB-specific response to PAH, consisting of the up-regulation of many genes involved in cell localization, negative regulation of cell death and angiogenesis. However, no apoptotic cells could be identified in the dataset, suggesting that apoptosis is not occurring at this stage in the SuHx model, in agreement with a peak of EC apoptosis occurring earlier, at 7 days.^5^ We revealed the CapillaryB-specific regulation of tip cell-enriched genes *Apln* and *Cd34*^40,^^41^ in both the mouse and human data, but without the detection of genuine tip cells. Interestingly, vessel regression, which is thought to be associated with dysfunctional sprouting angiogenesis in PAH,^4^ can occur via different processes, including intussusceptive angiogenesis^53^ or EC migration involving a tip cell phenotype as seen in zebrafish.^54^ More work is required to determine if any of these processes occur in PAH.^34^

We analysed zonation-dependent changes across the arteriovenous axis in PAH, confirming the continuum of transcriptional states, as previously described for brain ECs.^19^ The comparison between PAH and Control samples revealed specific gene regulation in distinct regions of the axis. In particular, *Sgk1* showed an up-regulation in ECs corresponding to arterioles/pre-capillary vessels, vasculature which is particularly affected by remodelling and neomuscularization in PAH.^7^ Since *Sgk1* regulates angiogenesis^55^ and *Sgk1* deficiency prevents hypoxia-induced PAH in mice,^56^*Sgk1* appears to be a key regulator of the primary changes occurring in ECs. Two extracellular matrix-associated protein in the SPARC family were also up-regulated, with a prominent up-regulation of *Sparc* in pre-capillary ECs and *Sparcl1* in capillary ECs. *Sparc* contributes to angiogenesis, with both pro-angiogenic and anti-angiogenic effect reported,^57^ while *Sparcl1* has recently been reported as a biomarker of maladaptive right ventricular remodelling in pulmonary hypertension.^58^

To identify promising EC gene targets relevant to the human disease, we compared the mouse PAH scRNA-seq with human genetic,^3^ and rat^21^ and human^22^ transcriptomic data. In addition to the down-regulation of *Bmpr2* gene, the main genetic driver of PAH, we showed up-regulation of *Aqp1* in ECs in PAH. The *Aqp1* knockout mouse has an attenuated response to hypoxia-induced PAH,^59^ suggesting *Aqp1* function in ECs contributes to PAH progression. Our transcriptomic comparison across models and species showed the relevance of this high-resolution mouse EC PAH analysis and highlighted novel candidates to modulate EC dysfunction in PAH. The cross-species analysis was also essential to define gene targets differentially regulated across species and in early and late stages of the disease. However, the human IPAH scRNA-seq^22^ analysis was limited by the small number of patient samples, preventing an analysis of patient variability. Future studies, including more human samples and additional time points in the rodent PAH models, are required to fully characterize PAH disease progression.

Among the candidates conserved across species, we focussed on *CD74*, as an increase in EC CD74 protein level has previously been identified in human PAH samples.^45^ CD74 is a receptor for the macrophage migration inhibitory factor, and the CD74/MIH complex was associated with PAH and linked to the recruitment of leucocytes to ECs *in vitro.*^45^ The scRNA-seq revealed that *Cd74* up-regulation is associated with changes to MHC-II genes, suggesting that the CD74/MHC-II complex might contribute to PAH progression. As multiple functions for CD74 have been reported,^46^ we expanded the functional characterization of *CD74* in ECs and showed its role in barrier function as well as proliferation, suggesting a potential role of CD74 in the hyperproliferative EC phenotypes characteristics of late PAH.

Overall, our study provides high resolution insights into the diversity of EC subpopulation responses to pulmonary hypertension and highlights novel candidates for future therapeutic development.

## Supplementary material

Supplementary material is available at *Cardiovascular Research* online.

## Author’s contributions

J.R., J.P.S., A.C., A.S., A.-M.S., and A.H.B. designed the experimental model. J.P.S., A.-M.S., J.P.M., A.C., K.R.S., A.S., R.D., B.E.P.H., K.S., P.W.F.H., S.-H.C., M.S., S.D.M., and P.D.U. contributed to the *in-vivo* work and sample preparation/tissue collection. J.R., S.-H.C., and S.S. performed the bioinformatics analysis. J.R., S.-H.C., L.P.M.H.d.R., and P.C. interpreted the bioinformatics data. M.S. performed and interpreted immunostaining of mouse tissue. J.P.M., Z.L., and M.B. designed the *in-vitro* experiments. J.P.M. performed the *in-vitro* experiments. S.Y.C., A.H., and R.L. provided the human scRNA-seq dataset. A.H.B., M.B., N.W.M., and N.C.H. supervised the research. A.H.B. secured funding. J.R., S.-H.C., J.P.S., and A.H.B. wrote the manuscript with input from all authors. All authors discussed the data and edited the manuscript.

## Supplementary Material

cvab296_Supplementary_Data

## Notes

This manuscript was handled by Consulting Editor Henning Morawietz.
