# Supplementary material for: Single-cell RNA sequencing profiling of mouse endothelial cells in response to pulmonary arterial hypertension
Source: Cardiovasc Res. 2021 Nov 15;118(11):2519–34. doi: 10.1093/cvr/cvab296 (PMC9400412; doi:10.1093/cvr/cvab296)
Supplement: cvab296_Supplementary_Data [file cvab296_supplementary_data.zip › Supplementary.Methods_Rodor_cvr_submission.2.docx]

**SUPPLEMENTARY METHODS**

**Single cell RNA-seq profiling of mouse endothelial cells in response to pulmonary hypertension**

Julie Rodor^1†^, Shiau-Haln Chen^1†^, Jessica P.Scanlon^1††^, João P. Monteiro^1††^, Axelle Caudrillier^1^, Sweta Sweta^1^**,** Katherine Ross Stewart^1^, Alena Shmakova^1^, Ross Dobie^2^, Beth E.P Henderson^2^, Kevin Stewart^1^, Patrick W.F. Hadoke^1^, Mark Southwood^3^, Stephen D. Moore^3^,Paul D. Upton^3^, Nick W. Morrell^3^, Ziwen Li^1^, Stephen Y. Chan^4^, Adam Handen^4^, Robert Lafyatis^4^, Laura P. M. H. de Rooij^5^, Neil C. Henderson^2^, Peter Carmeliet^5^, Ana-Mishel Spiroski^1^, Mairi Brittan^1^, Andrew H. Baker^1*^

**Induction of pulmonary hypertension in Cdh5-CreERT2 mouse**

The *Cdh5-CreERT2* mouse line was obtained using the Tg(Cdh5-cre/ERT2)1Rha transgene (Ralf H Adams, MGI:3848982), corresponding to the insertion of the tamoxifen-inducible cre/ERT2 sequence into a P1 artificial chromosome containing the *Cdh5* gene. Control female mice were either euthanised immediately following completion of tamoxifen induction or held in standard housing conditions in parallel with female mice undergoing pulmonary hypertension procedure. After 2-week tamoxifen washout, we induced pulmonary hypertension in 10-week old mice as previously described^1, 2^. Briefly, mice were injected with 20 mg/kg SU5416 weekly for three weeks while exposed to hypobaric hypoxia (10%). 4mg/ml SU5416 was suspended in 0.9% sterile saline with 0.5% carboxymethylcellulose sodium [w/v], 0.4% polysorbate 80 [v/v] and 0.9% benzyl alcohol [v/v] by sonication. Mice were housed in hypobaric hypoxia, maintained in a plexiglass chamber with nitrogen gas autoregulated to provide 10% oxygen. Ammonia was removed by ventilation and activated charcoal filtration.

Upon completion of the pulmonary hypertension procedure, or 3-weeks holding in Control mice, right ventricular systolic pressure (RVSP) was measured under terminal isoflurane anaesthesia. Briefly, anaesthesia was induced with 4% isoflurane in 2.0 ml/min supplemental oxygen and maintained at 1.5-2.0% with homeothermic support (PhysiTemp, ADInstruments) throughout the procedure. The right jugular vein was exposed and a Millar solid state catheter (SPR-671NR, ADInstruments) advanced into the right ventricle. After stabilisation, 10-minute pressure recordings were collected through a BridgeAmp on a PowerLab 4/26 in LabChartPro (ADInstruments) and RVSP was determined. Mice were euthanised by exsanguination.

Hearts were micro-dissected and ventricular weights collected. Right ventricular hypertrophy was calculated as right ventricle (RV) weight divided by the sum of the left ventricle with septum (LV+S).

**Induction of pulmonary hypertension in C57/BL6 mouse and tissue collection**

Female C57/BL6 mice at 10 weeks of age (~19–22g, Charles River) were used. One group (n=9) was injected with subcutaneous injections of SU5416 (20 mg/kg; Tocris, Bristol, UK) in vehicle (0.5% carboxyl methylcellulose sodium, 0.4% polysorbate 80, 0.9% benzyl alcohol; all Sigma), placed immedi­ately into a 10% O2 chamber and maintained in hypoxia for 3 weeks. These animals were briefly removed from hypoxia at the end of weeks 1 and 2 for administration of second and third doses of SU5146, respectively. A second group (n=5) was maintained in normoxia. At the end of the 3-week period, mice were anaesthetised with isofluorane and right ventricular pressures and volumes were recorded using a Millar SPR-839 catheter (Millar Instruments, Houston, TX). Mice were then sacrificed and the hearts and lungs were harvested. Right ventricular hypertrophy was determined as the ratio of right ventricular to left ventricular and septal weight (RV/LV+ S). The right lungs were tied off with suture and the left lung inflated and fixed with 10% neutral-buffered formalin for 5 minutes prior to excision and immersing in 10% neutral-buffered formalin (CellPath, Newtown, Powys, UK), prior to dehydration and paraffin embedding.

**Pulmonary arteriolar muscularisation analysis**:

Sections of formalin fixed paraffin wax mouse lung tissue (4μm) were labelled with monoclonal mouse-anti-smooth muscle α-actin (clone 1A4, Agilent, UK) and detected using an Animal Research Kit^™^ peroxidase (ARK^™^, Agilent, UK) following the manufacturer’s instructions. Briefly, the primary antibody was labelled using a modified biotinylated anti-mouse immunoblobulin prior to application to the specimen. The primary antibody and Biotinylation Reagent were mixed in solution, resulting in binding of biotinylated secondary antibody to the primary antibody. The blocking reagent (containing mouse serum) was then added to the mixture to bind residual Biotinylation Reagent not bound to the primary antibody, minimizing potential interaction with immunoglobulin endogenous in the specimen. The biotin-labelled primary antibody was then applied to the specimen followed by incubation with streptavidin-peroxidase, visualised using 3, 3’- diaminobenzidine (DAB) to create a brown reaction product and counterstained with Haematoxylin (TCS Bioscience, UK).

Assessment of pulmonary arteriolar muscularisation involved the identification of alveolar ducts and the subsequent categorization of the accompanying intra-acinar artery as non-, partially- or fully-muscularised. An average of n=20.8 (range 7-41) vessels from lung sections were categorised as either fully, partially or non-muscularised. Statistical significance was assessed by comparing the percentage of fully muscularised vessels between groups. Assessment of muscularisation was performed blinded.

**Isolation of TdTomato^+^ cells from mouse lung**

TdTomato^+^ mouse lung cells were isolated as previously described^3^. In brief, the thoracic cavity was opened, to allow access to the lungs. Then a tracheotomy was performed, and 1ml of Liberase (SigmaAldrich, Paisley, UK) (prepared in PBS-EDTA with 1mg/ml of Liberase^TM^ buffer and 1U DNase) injected through the trachea into the lungs. The trachea was then tied off, and the lungs removed and placed into an Eppendorf. Lungs were then incubated for 20min at 37°C in a shaking water bath and further processed using a gentleMACS Dissociator (Miltenyi Biotec, Auburn, CA). After digestion, the lung-enzyme mix was filtered through a 70μm nylon mesh (Pre-Separation Filters, 70μm, #130-095-823) and centrifuged at 2000 rpm for 5 minutes at 4°C. The pellet was resuspended in 5ml of ACK lysis buffer (ThermoFisher, Paisley, UK) and left for 5 minutes on ice, according to manufacturer’s protocol. The cells were then centrifuged at 2000 rpm for 5 minutes. Cells suspension was incubated with PBS-1% BSA for 15 minutes and then separated using the BD FACSAria II cell sorter based their TdTomato^+^ status. Single cells (singlets) were selected first using forward scatter area (FSC-A) versus forward scatter height (FSC-H), followed by side scatter area (SSC-A) versus side scatter height (SSC-H). From the singlet population, viable (live) cells were selected by gating for DAPI negative events. Total lung cells were then separated from debris based on size and granularity by plotting forward versus side scatter. ECs were finally selected by gating for TdTomato^+^ events compared to control samples.

**Mouse bulk RNA-seq analysis:**

RNAs isolation from TdTomato^+^ cells of 5 Control (b_Cont1-5) and 4 PAH (b_PAH1-4) samples were carried out using the Qiagen miRNeasy Micro Kit. PolyA stranded libraries were prepared by Genewiz using SMARTseq HT Ultra-Low kit (unstranded). Libraries were sequenced with HiSeq 4000 at an average of 44.6 million reads per sample (paired end, 2 x 150bp).  Quality and adapter read trimming was performed using Trim Galore version 0.5.0. Gene quantification (read count and normalised expression value as Fragments Per Kilobase Million - FPKM) was obtained using RSEM version 1.3.0 (options: -bowtie2 --p 20 --paired-end), based on GENCODE GRCm38 genome primary assembly with annotation version M16.

The differential expression was assessed using DESeq2 version 1.26.0 by comparing PAH to Control samples. We considered a threshold of absolute Fold Change >=1.5 and adjusted pvalue<0.05 to identify significant changes between two conditions. We also applied an expression value threshold of at least 2 FPKM in 2 samples. Expression of selected genes was displayed as a heatmap of the z-score of the Log2(FPKM+1) using the package pheatmap version 1.0.12.

**Mouse Single-cell RNA-seq analysis:**

**Library preparation**

Single cell RNA-sequencing libraries were prepared using the Single Cell 3′ Reagent Kit User Guide v2 (10x Genomics) following manufacturer’s protocol. In brief, single cells suspensions were loaded on a Chromium Controller instrument. Following generation of the single-cell gel bead-in-emulsions (GEMs), GEM-reverse transcriptions (GEM-RTs) was performed. After RT, GEMs were harvested and the cDNAs were amplified and cleaned up with the SPRIselect Reagent Kit (Beckman Coulter). Indexed sequencing libraries were constructed using the Chromium Single-Cell 3′ Library Kit (10x Genomics). This protocol included enzymatic fragmentation, end-repair, A-tailing, adaptor ligation, ligation clean-up, sample index PCR, and PCR clean-up. Libraries were sequenced on NovaSeq S2 at Edinburgh Genomics.

**Pre-processing analysis**

Read mapping and generation of the expression matrix was done using CellRanger version 3.1.0 using ‘cellranger count’ function on a custom annotation. The custom annotation was created using ‘cellranger mkref’ command based on mm10 Cell Ranger reference package 3.0.0 and including the transcript sequence of TdTomato (CDS+ 3’UTR). Subsequent analysis was done using R version 3.6.3. Low quality cells were removed independently from each dataset using scater version 1.14.6^4^. Cell-level and gene-level metrics were calculated using the perCellQCMetrics and addPerFeatureQC functions respectively. Cells that were more than 3 median absolute deviation from the median of the quality control metrics (total counts, number of genes detected and percentage of mitochondrial genes) were identified as low-quality and they were discarded from downstream processing and analyses.

**Analysis of ‘merge’ data**

Following data normalisation to remove unwanted sources of heterogeneity using batchelor^5^ version 1.2.4, all samples from both control and PAH were then merged and converted into a single Seurat object. Downstream analysis was performed using Seurat^6^ version 3.1.5, unless otherwise stated. The top 2000 most variable genes were identified using the 'vst’ method in the FindVariableFeatures function. All genes were then scaled and centred, and principal component analysis was performed using the previously identified variable genes. The data’s elbow plot was used to determine the number of principal components (PCs) to include. For experiment 1 (ContA & ContB), 4 PCs were kept for clustering, and 7 PCs were kept for the clustering of all samples. All clustering was performed at a resolution of 0.1. Seurat’s graph-based clustering method was applied and the data was visualised using Uniform Manifold Approximation and Projection (UMAP). The identities of cells with low *TdTomato* expression were confirmed using SingleR^7^ version 1.0.6 and the in-built MouseRNAseqData reference dataset (SnapshotDate: 2019-10-22). This dataset contains 358 mouse RNA-sequencing samples with annotations for 18 main cell types^8^. Non-endothelial cells identified by SingleR were removed from integration-based downstream analyses, keeping 24,333 cells out of 25,357 cells.

**Identifying TdTomato^+^/mesenchymal^+^ cells:**

TdTomato+ cells were defined and selected using an expression value of *TdTomato* greater than 0.2 (function WhichCells and subset from Seurat). Acta2^+/-^ and Col1a1^+/^- were subsequently defined based on an expression threshold of 0.2.

**Analysis of ‘integrated’ data**

In order to correct for batch effects present across the two experiments and within each replicates, we applied the standard integration workflow from Seurat. The top 2000 variable features were first identified for each sample independently. Anchors across the dataset were then determined and used to integrate the chosen samples using the default 30 dimensions. Standard clustering and visualisation steps were then performed on the integrated assay. Clustering resolution was kept at 0.1 and the number of PCs included for clustering were: 6 for Control only, 7 for PAH only and 8 for all samples.

**Cell Cycle analysis:**

Cell cycle scoring was performed using the function CellCycleScoring from Seurat. Markers of G2/M and S phases were obtained from Seurat (based on Tirosh et al, 2015) and converted to mouse gene nomenclature.

**Differential gene expression**

Differential gene expression analysis was performed by using the function FindMarkers from Seurat on the “RNA” assay and by comparing between Control and PAH conditions (LogFC of 0.25 and min.pct=0.2). To obtain a stringent differential expressed gene set, we first carried out independent analysis for each PAH mouse, keeping the common changes compared to the individual Control. Then, we focused on changes common to PAH1, PAH2 and PAH3. This approach was used to study the changes across the arteriovenous axis.

**Analysis of CapillaryB Specific gene response.**

Hierarchical clustering of the CapillaryB DGEs was obtained using pheatmap package version 1.0.12 on the scaled data from the Seurat object, using a downsampling of 500 cells per category. The tree was cut in 3 using the function “cutree” and CapillaryB specific response genes, corresponding to cluster 1, was retrieved.

**Pathway and Gene Ontology analysis**

The analysis was done on the DEGs obtained in each EC populations between PAH and Control (pooled data). As a background, we used the scRNA-seq detectable genes. Enriched KEGG pathway was obtained using the function enrichKEGG from the package ClusterProfiler^9^ version 3.14.3. The visualisation of the DEGs on relevant pathway graph was obtained using pathview^10^ version 1.26.0. Gene ontology analysed was done using topGO version 2.38.1. Signature score of genes associated with PAH-relevant Go terms was obtained using the AddModuleScore function of Seurat.

**Defining and ordering cells across the arteriovenous axis**

Cells corresponding to Artery, CapillaryA and Vein situated in the same part of the UMAP plot were selected (function subset and CellSelector from Seurat). The identification of the lineage/axis structure was carried out using Slingshot^11^ version 1.4.0 based on the Integrated assay. The “pseudotime” information corresponding to the position along the axis was extracted. For differential gene expression analysis, the axis was divided in 10 sections and a “pooled” as well as a stringent PAH versus Control comparison was performed. The heatmap showing the log fold change of the “pooled” analysis was plotted for all stringent DEGs across the axis using the package pheatmap version 1.0.12. The expression profile across the axis was obtained with the function geom_smooth() from ggplot2 using method = 'gam' and formula 'y ~ s(x, bs = "cs").

**Rat Single-cell RNA-seq analysis:**

The expression matrix (ln(x+1)) from Hong et al, 2020 was downloaded from the following link <http://mergeomics.research.idre.ucla.edu/PVDSingleCell/>. After conversion to a Seurat object, endothelial cells were retrieved based on Hong et al annotation ("Endothelial arterial 1","Endothelial arterial 2", "Endothelial capillary"). This data was then integrated to the mouse dataset (Seurat object containing EC only and integration of all individual samples) using the integration command from Seurat (FindIntegrationAnchors and IntegrateData). Downstream processing and clustering were done as described above for the mouse data (using 7 dimensions and 0.1 resolution). Clusters were annotated based on mouse markers and the expression of similar markers in the rat cells was confirmed. Differential expression analysis was carried out in rat Artery, Vein, CapillaryA and CapillaryB using FindMarkers function of Seurat. SuHx samples and MCT samples were compared to Control samples, after pooling all replicates. Due to the low number of cells, significant changes were obtained based on a pvalue threshold of 0.05 (without multiple comparisons), a logfc threshold of 0.25 and a min.pct of 0.2.

**Human Single-cell RNA-seq analysis:**

Raw sequencing data from human Control/IPAH scRNA-seq published in Saygin et al, 2020^12^ was obtained by contacting the authors of the manuscript. Sample1 (Control 76yo)^12^ was not included due to its low cell numbers. Read mapping and generation of the expression matrix was done using CellRanger version 3.1.0 using ‘cellranger count’ function on the GRCh38-3.0.0 annotation downloaded from the 10X Genomics website. Subsequent analysis was done using R version 3.6.3. Similarly to the mouse scRNA-seq data analysis, cell filtering was done using scater version 1.14.6^4^ and data normalisation using batchelor^5^ version 1.2.4. Downstream analysis was performed using Seurat^6^ version 3.1.5. After merging all samples, dimensional reduction and clustering was performed (with 12 dimensions and 0.1 resolution). Cluster corresponding to endothelial cells was identified based on the high expression of the endothelial markers, including *CDH5*, *PECAM1* and *CLDN5*. To identify endothelial cell subpopulations, we analysed ECs separately. To take into account patient variability, we performed an integration of individual samples using Seurat (k.filter was set up to 45 to allow the integration of the samples with the lowest number of ECs). Following integration, dimensional reduction and clustering was performed using 8 dimensions and a resolution of 0.1. Differential expression analysis was carried out in the Arterial, Vein, Capillary A and Capillary B subpopulations. Significant changes were identified using a p-adj threshold of 0.05, absolute logfc of 0.25 and min.pct of 0.2. Analysis of the relative proportion of EC subpopulations across conditions were performed after removing samples Control 2 and Control 3 with low number of ECs.

**Comparison across species:**

We compared the list of DEGs identified in each species by simply looking for the same gene name across species. For MHC-I and MHC-II, the names and number of genes are different between rodent and human, so we considered an overlap between species if we found members from the same class of genes, based on comparative genomics study of human and mouse histocompatibility complex^13^.

**Human umbilical vein endothelial cell culture and transfection:**

Pooled donors HUVEC #C2519A were purchased from Lonza (Basel, Switzerland) and maintained in endothelial cell growth medium (EGM-2 BulletKit™) (Lonza, Basel, Switzerland) supplemented with foetal bovine serum (FBS) (10%, Life Technologies, Paisley, UK) and Penicillin-Streptomycin (P/S) (100U/ml) (Gibco, Paisley, UK). HUVEC were used between P3 and P8, and kept at 37°C in a humidified atmosphere containing 5% CO_2_.

All transfections were performed in 1ml of EGM-2 with cells at 70% confluence cultured in 6-well collagen-coated plates. Both *CD74* (siCD74: ID: s2714) and negative control (siControl: ID 4390843) siRNA (Ambion) were transfected at 10nM using lipofectamine RNAiMAX Transfection Reagent (Life Technologies, Paisley, UK) according to the manufacturer’s instructions (See Table 1for list of reagents). The transfection medium was replaced with EGM-2 after 24h and downstream analysis carried out after 72h.

**Proliferation analysis of HUVEC**

Click-iT™ Plus EdU Alexa Fluor™ 488 Flow Cytometry Assay Kit (Invitrogen) was used according to manufacturer’s instructions to measure HUVEC proliferation. Cells were counted and replated in equal numbers 24h prior harvest. Cells were incubated with 10 uM EdU for a total of 24h and later trypsinised, fixed and permeabilised. EdU incorporation was quantified using Click-it chemistry conjugated with an Alexa Fluor 488 antibody according to the manufacturer's protocol. He stained samples were then acquired on a BD LSRFortessa™ cell analyser. Histograms and analysis were obtained using FlowJo software.

## HUVEC barrier integrity assay:

The Electric Cell-substrate Impedance Sensing (ECIS) assay was implemented using 8-well 8W10E+ arrays via a ECIS® Z-Theta station (Applied Biophysics), as per the manufacturer’s instructions. All arrays were treated using 10 mM L-cysteine (C7352-25G, Sigma-Aldrich) and coated with 0.2% Gelatine (Sigma-Aldrich) and 10ug/mL fibronectin (Sigma-Aldrich). A total of 5x10^4^ HUVEC were seeded with EGM-2 into each well of the array and allowed to adhere overnight. The assay was then conducted using a monitoring frequency of 4 kHz for a total of 6 h. All impedance measurements, expressed as electrical resistance (Ohm), were further analysed using ECIS mathematical modelling software (Applied Biophysics) to determine cell-cell interaction, expressed as Rb (Ohm x cm^2^), and cell-matrix interaction, expressed as Alpha (cm x Ohm^0.5^).

**Statistical analysis:**

Statistical analysis was performed using GraphPad Prism 9.0.0 or R version 3.6.3.

For mouse measurements and tissue analysis (RVSP, hypertrophy, vessel remodelling, Ki67/CD31 staining) with n>=5, normal distribution was tested using the Shapiro-Wilk test. For data following a normal distribution, significant differences between conditions were tested using an unpaired 2-tailed t-test. For data not following a normal distribution, significant differences between conditions were tested using a Mann-Whitney test.

For the scRNA-seq analysis, significance of differential expression changes was determined using the default option of FindMarkers function of Seurat that relies on a Wilcoxin Rank Sum test with adjusted p-value obtained based on Bonferroni correction using all genes in the dataset. Analysis of difference in terms of relative EC population proportion and cell cycle phase was done on the Log2 of the percentage using an unpaired t-test.

For *in-vitro* experiments in HUVEC, as each experimental data set is an average of a large number of cultured cells, we assumed the data was normally distributed based on the central limit theorem. Statistical analysis between siCD74 and siCTR samples was performed using an unpaired t-test.

**Plots**

Plots were produced using the ggplot2 package alone, using Seurat functions in conjunction with ggplot2 or GraphPad Prism 9.0.0. FeaturePlot was used with parameters: min.cutoff = "q10", max.cutoff = "q90".

**Data availability**

We have made our data accessible for further exploration through an interactive web app http://bakergroup.shinyapps.io/mouse_ec_pah, built using the Shiny package version 1.5.0.

**References for Supplementary Methods**

1. Vitali SH, Hansmann G, Rose C, Fernandez-Gonzalez A, Scheid A, Mitsialis SA, Kourembanas S. The Sugen 5416/hypoxia mouse model of pulmonary hypertension revisited: long-term follow-up. *Pulm Circ* 2014;**4**:619-629.

2. Deng L, Blanco FJ, Stevens H, Lu R, Caudrillier A, McBride M, McClure JD, Grant J, Thomas M, Frid M, Stenmark K, White K, Seto AG, Morrell NW, Bradshaw AC, MacLean MR, Baker AH. MicroRNA-143 Activation Regulates Smooth Muscle and Endothelial Cell Crosstalk in Pulmonary Arterial Hypertension. *Circ Res* 2015;**117**:870-883.

3. Fehrenbach ML, Cao G, Williams JT, Finklestein JM, Delisser HM. Isolation of murine lung endothelial cells. *Am J Physiol Lung Cell Mol Physiol* 2009;**296**:L1096-1103.

4. McCarthy DJ, Campbell KR, Lun AT, Wills QF. Scater: pre-processing, quality control, normalization and visualization of single-cell RNA-seq data in R. *Bioinformatics* 2017;**33**:1179-1186.

5. Haghverdi L, Lun ATL, Morgan MD, Marioni JC. Batch effects in single-cell RNA-sequencing data are corrected by matching mutual nearest neighbors. *Nat Biotechnol* 2018;**36**:421-427.

6. Stuart T, Butler A, Hoffman P, Hafemeister C, Papalexi E, Mauck WM, 3rd, Hao Y, Stoeckius M, Smibert P, Satija R. Comprehensive Integration of Single-Cell Data. *Cell* 2019;**177**:1888-1902 e1821.

7. Aran D, Looney AP, Liu L, Wu E, Fong V, Hsu A, Chak S, Naikawadi RP, Wolters PJ, Abate AR, Butte AJ, Bhattacharya M. Reference-based analysis of lung single-cell sequencing reveals a transitional profibrotic macrophage. *Nat Immunol* 2019;**20**:163-172.

8. Benayoun BA, Pollina EA, Singh PP, Mahmoudi S, Harel I, Casey KM, Dulken BW, Kundaje A, Brunet A. Remodeling of epigenome and transcriptome landscapes with aging in mice reveals widespread induction of inflammatory responses. *Genome Res* 2019;**29**:697-709.

9. Yu G, Wang LG, Han Y, He QY. clusterProfiler: an R package for comparing biological themes among gene clusters. *OMICS* 2012;**16**:284-287.

10. Luo W, Brouwer C. Pathview: an R/Bioconductor package for pathway-based data integration and visualization. *Bioinformatics* 2013;**29**:1830-1831.

11. Street K, Risso D, Fletcher RB, Das D, Ngai J, Yosef N, Purdom E, Dudoit S. Slingshot: cell lineage and pseudotime inference for single-cell transcriptomics. *BMC Genomics* 2018;**19**:477.

12. Saygin D, Tabib T, Bittar HET, Valenzi E, Sembrat J, Chan SY, Rojas M, Lafyatis R. Transcriptional profiling of lung cell populations in idiopathic pulmonary arterial hypertension. *Pulm Circ* 2020;**10**.

13. Shiina T, Blancher A, Inoko H, Kulski JK. Comparative genomics of the human, macaque and mouse major histocompatibility complex. *Immunology* 2017;**150**:127-138.
