# Supplementary material for: Single-cell RNA sequencing profiling of mouse endothelial cells in response to pulmonary arterial hypertension
Source: Cardiovasc Res. 2021 Nov 15;118(11):2519–34. doi: 10.1093/cvr/cvab296 (PMC9400412; doi:10.1093/cvr/cvab296)
Supplement: cvab296_Supplementary_Data [file cvab296_supplementary_data.zip › Supplementary.Figures_Rodor_cvr_submission.2.pdf]

Supplementary Figure S1

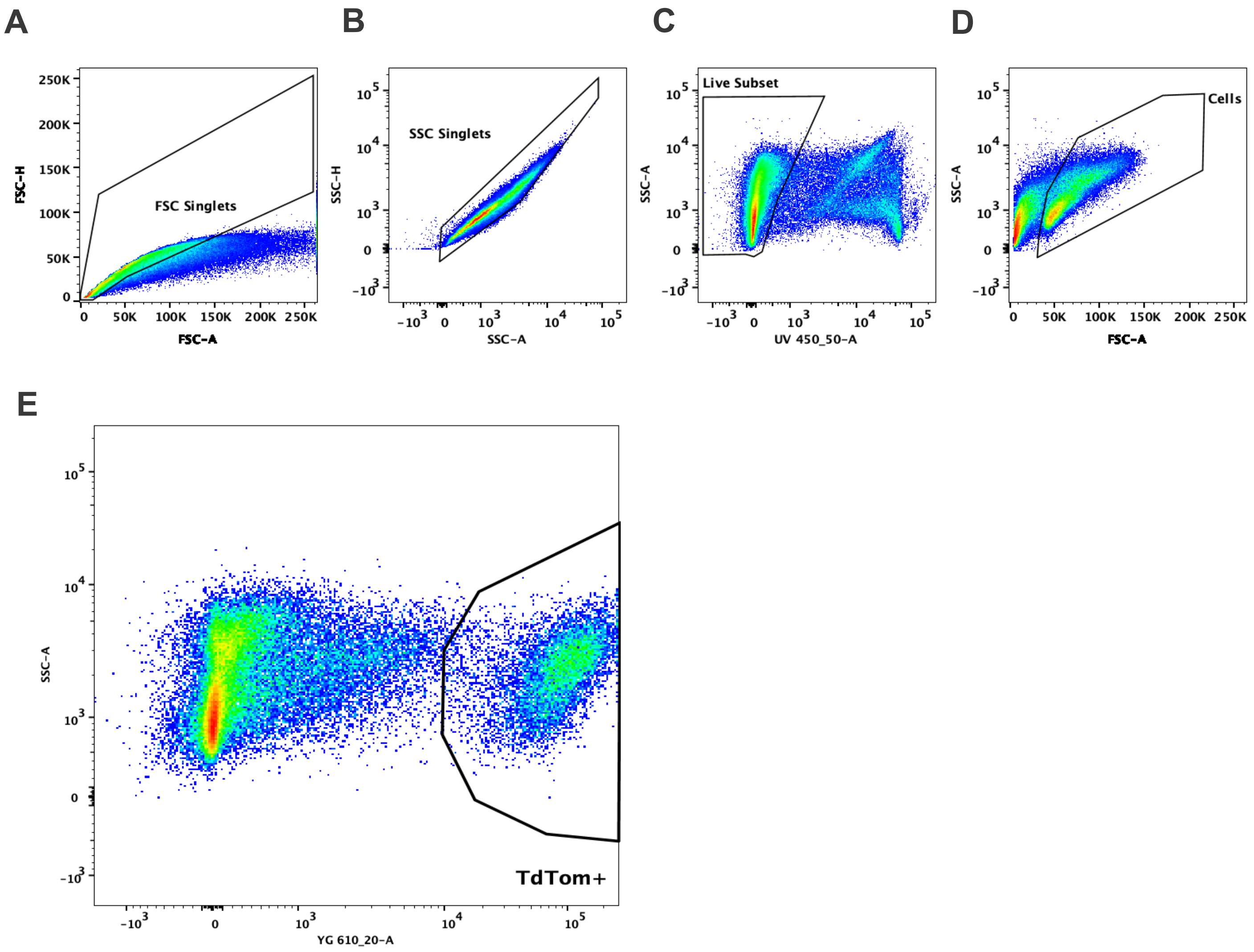

Supplementary Figure S2

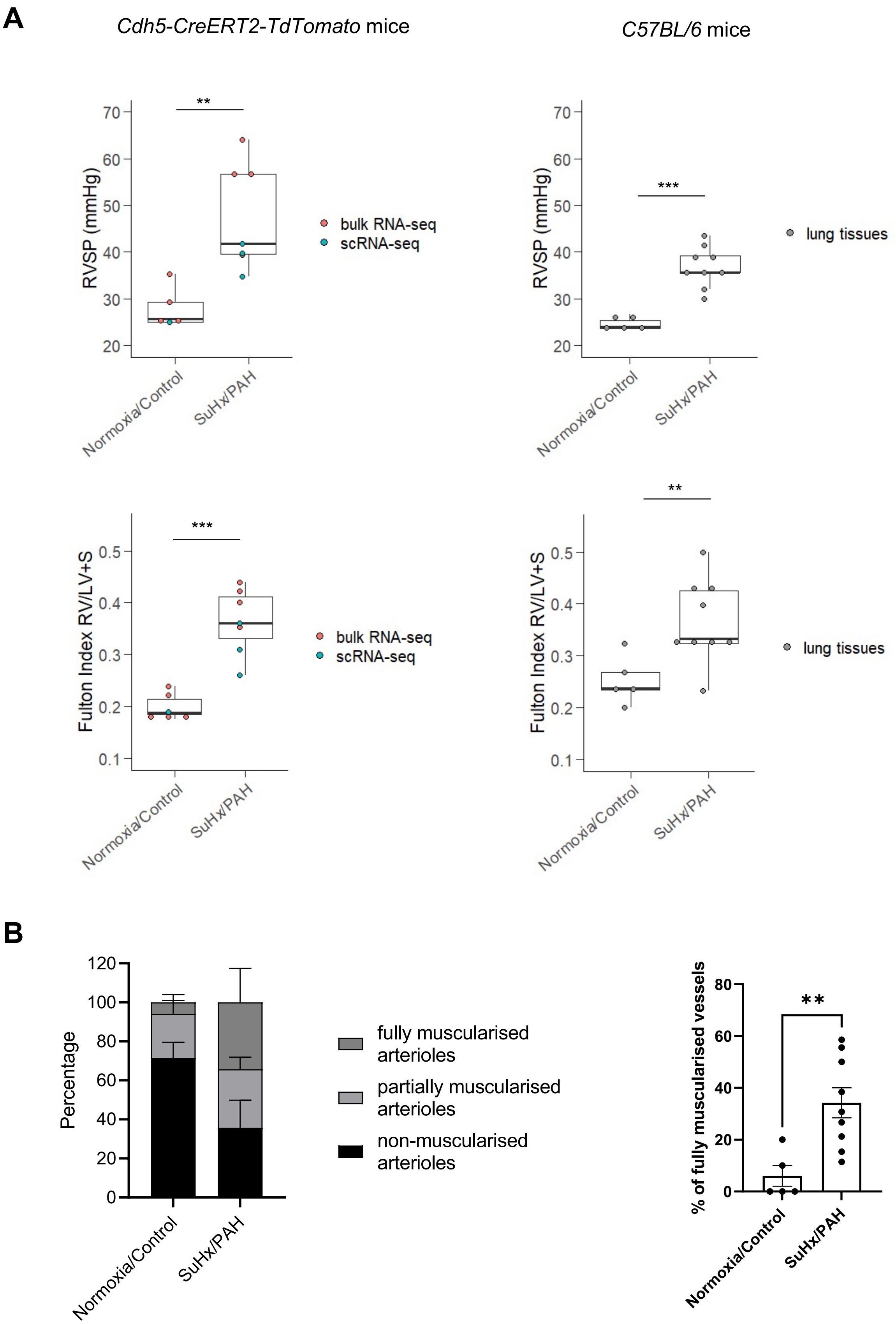

Supplementary Figure S3

A

	ContA	ContB	Average
Number of reads	357,347,266	368,877,140	363,112,203
Sequencing saturation	96.40%	95.40%	95.90%
Estimated number of cells	3,577	3,665	3,621
Mean reads per cells	99,901	100,645	100,273
Median genes per cell	770	1,018	894
Total genes detected	16,625	16,911	16,768
Median UMI counts per cells	1,279	1,769	1,524
Outlier cells based on mitochondrial contents	103 (2.9%)	425 (11.6%)	264
Filtered cells	184 (5.1%)	469 (12.8%)	327

B

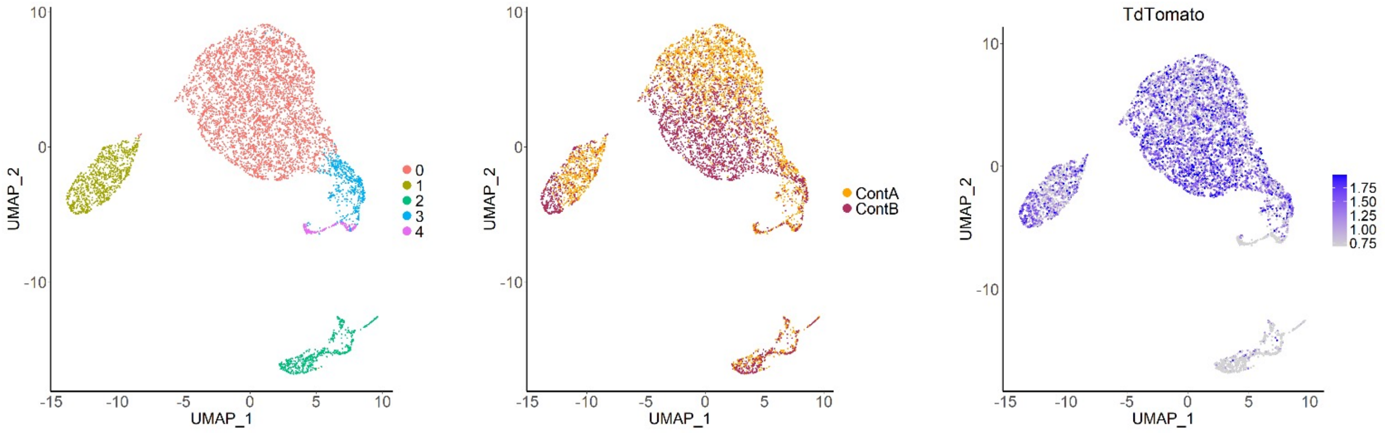

Supplementary Figure S4

	Cont1	PAH1	PAH2	PAH3	Average
Number of reads	593,763,486	501,542,278	601,642,821	675,558,973	593,126,890
Sequencing saturation	95.80%	93.30%	93.70%	98.30%	95.30%
Estimated number of cells	5,288	5555	6,310	3,162	5,079
Mean reads per cells	112,285	90287	95,348	213,649	127,892
Median genes per cell	983	1380	1,335	928	1,157
Total genes detected	17,286	17543	18,001	15,404	17,059
Median UMI counts per cells	1,701	2424	2,352	1,522	2,000
Outlier cells based on mitochondrial contents	314 (5.9%)	345 (6.2%)	394 (6.2%)	150 (4.7%)	301
Filtered cells	394 (7.5%)	436 (7.8%)	547 (8.7%)	170 (5.4%)	387

Supplementary Figure S5

A

	Cluster0	Cluster1	Cluster2	Cluster3	Cluster4	Cluster5	Cluster6	Cluster7
TdTomato expression	4.41	3.49	4.01	3.80	0.74	3.54	0.34	2.35
Number of cells	10221	8287	2632	2270	833	609	309	196

B

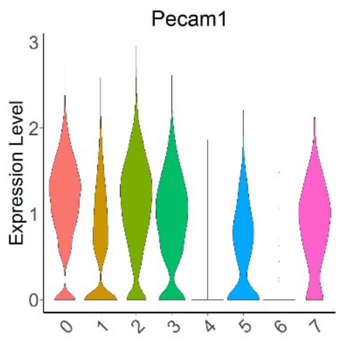

C

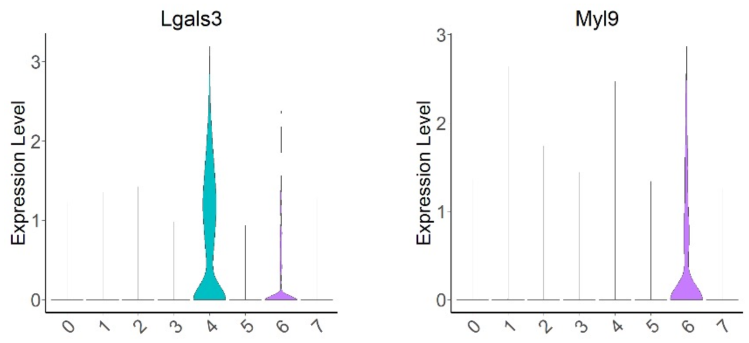

Supplementary Figure S6

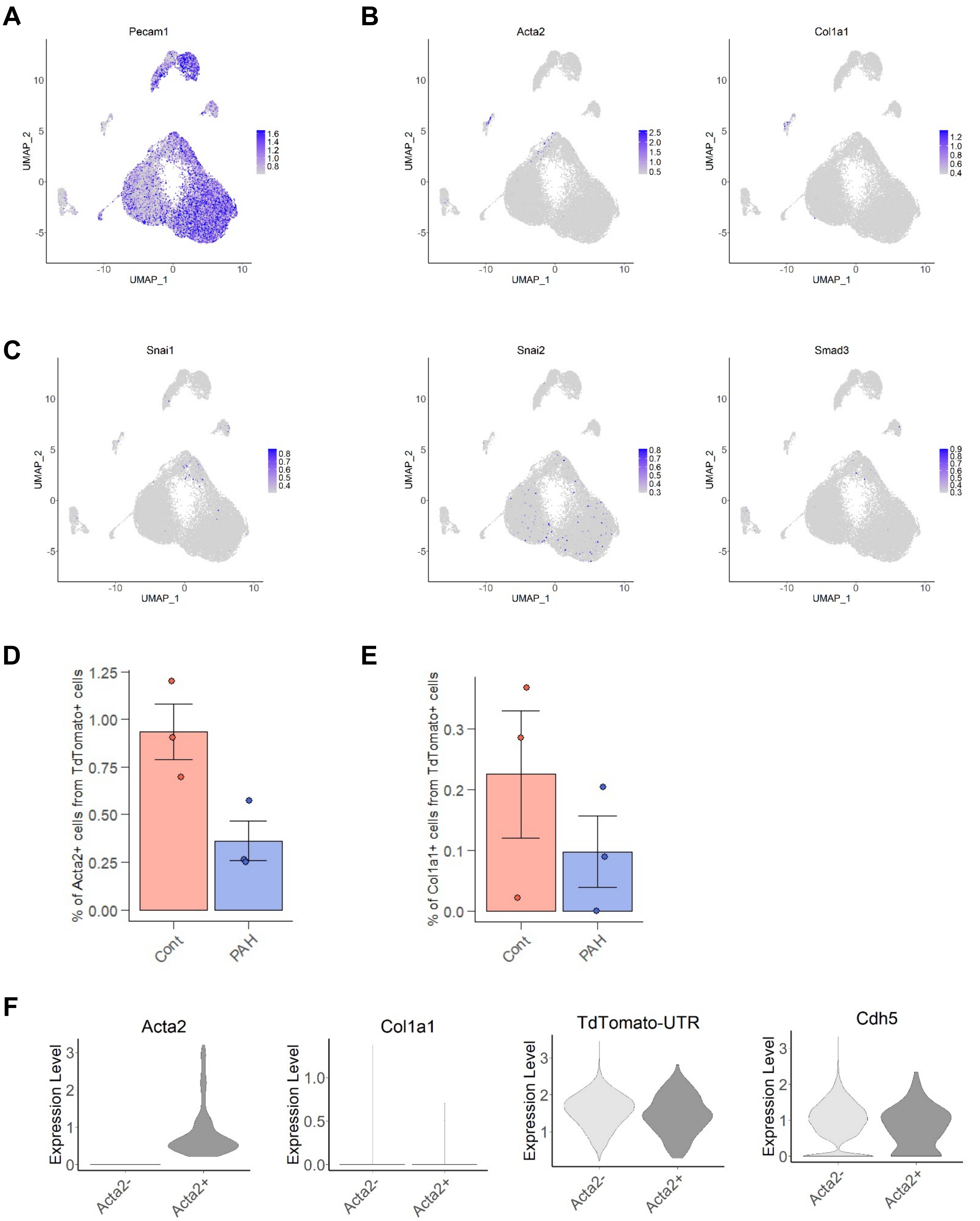

Supplementary Figure S7

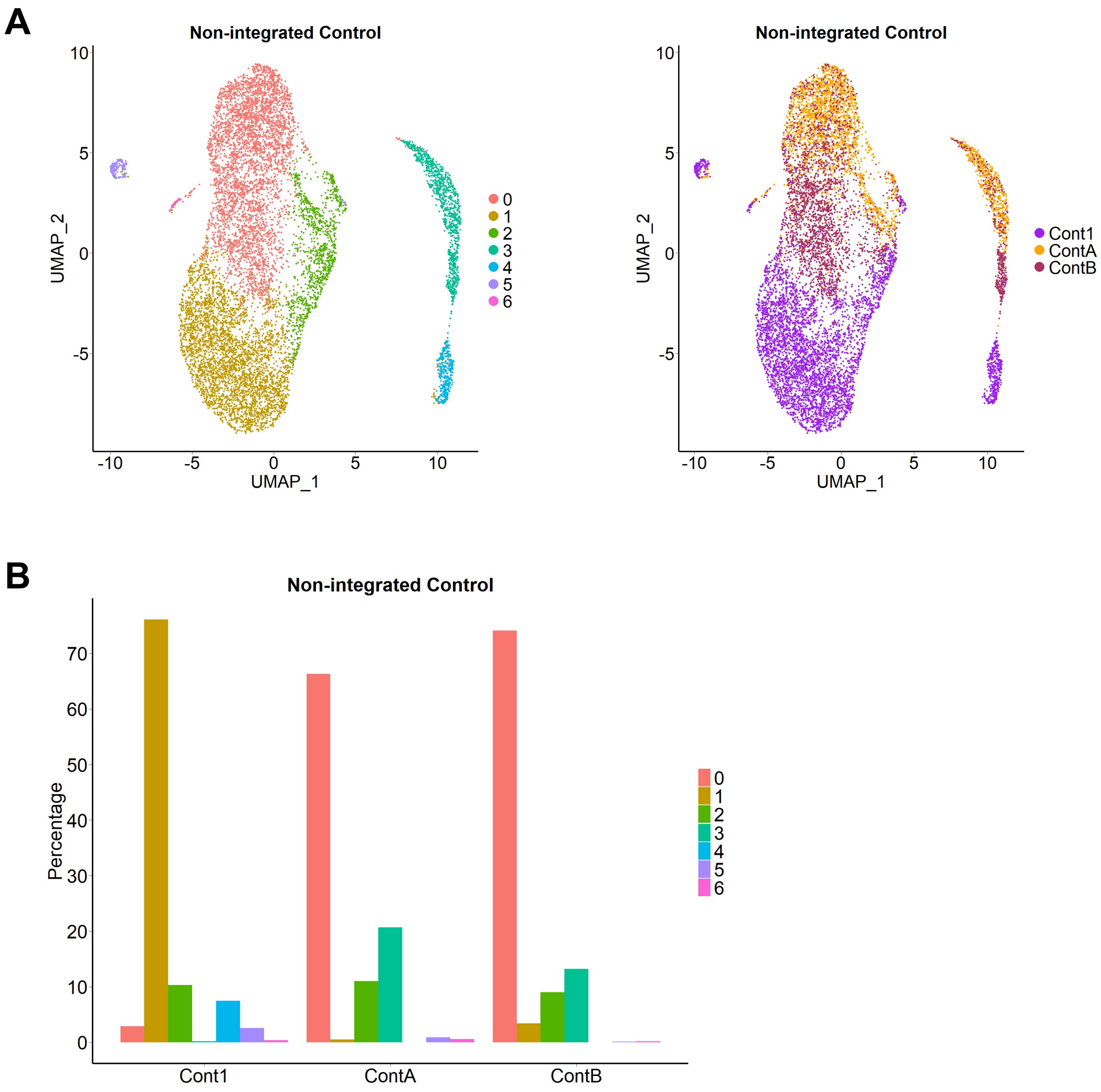

Supplementary Figure S8

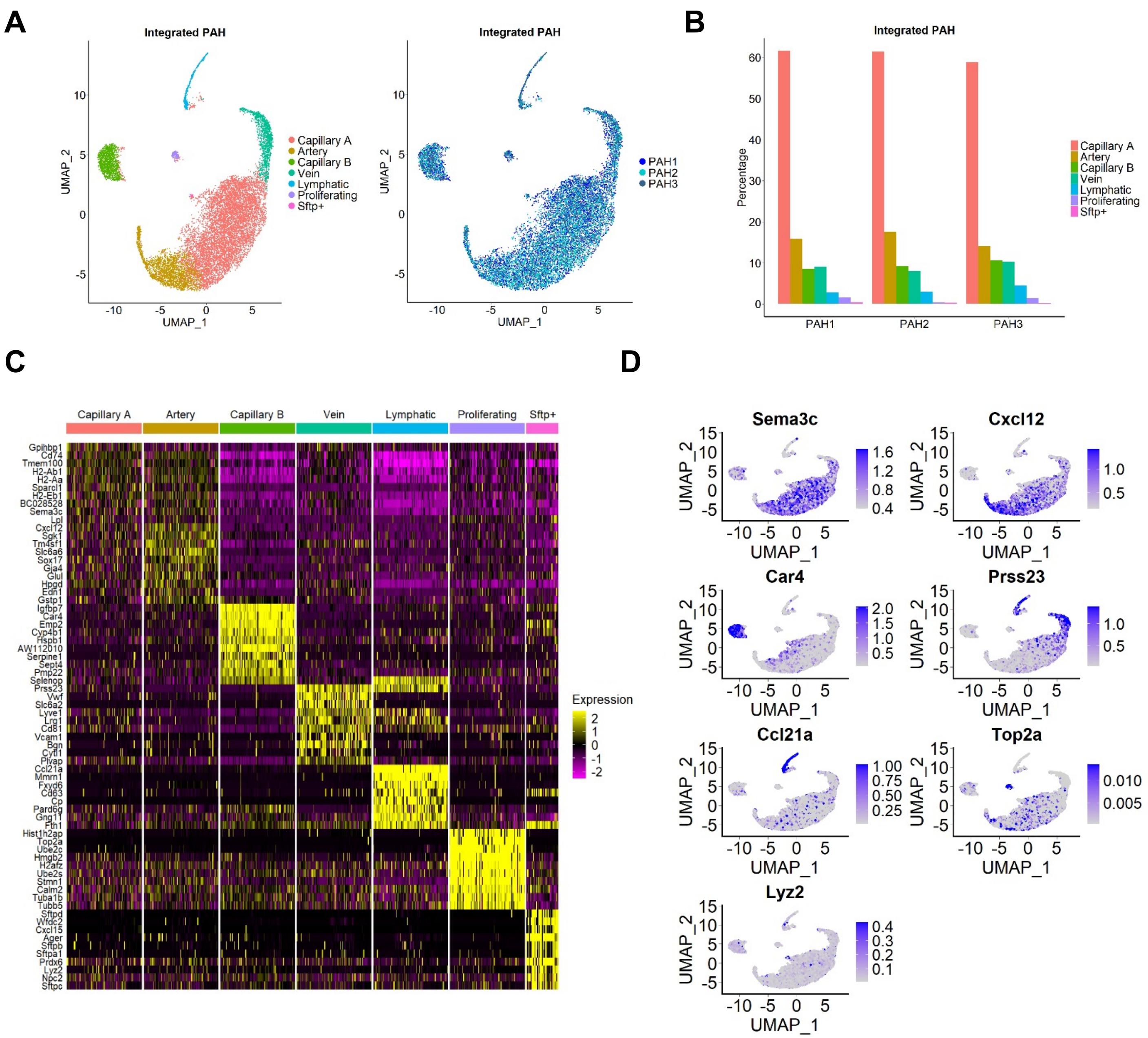

Supplementary Figure S9

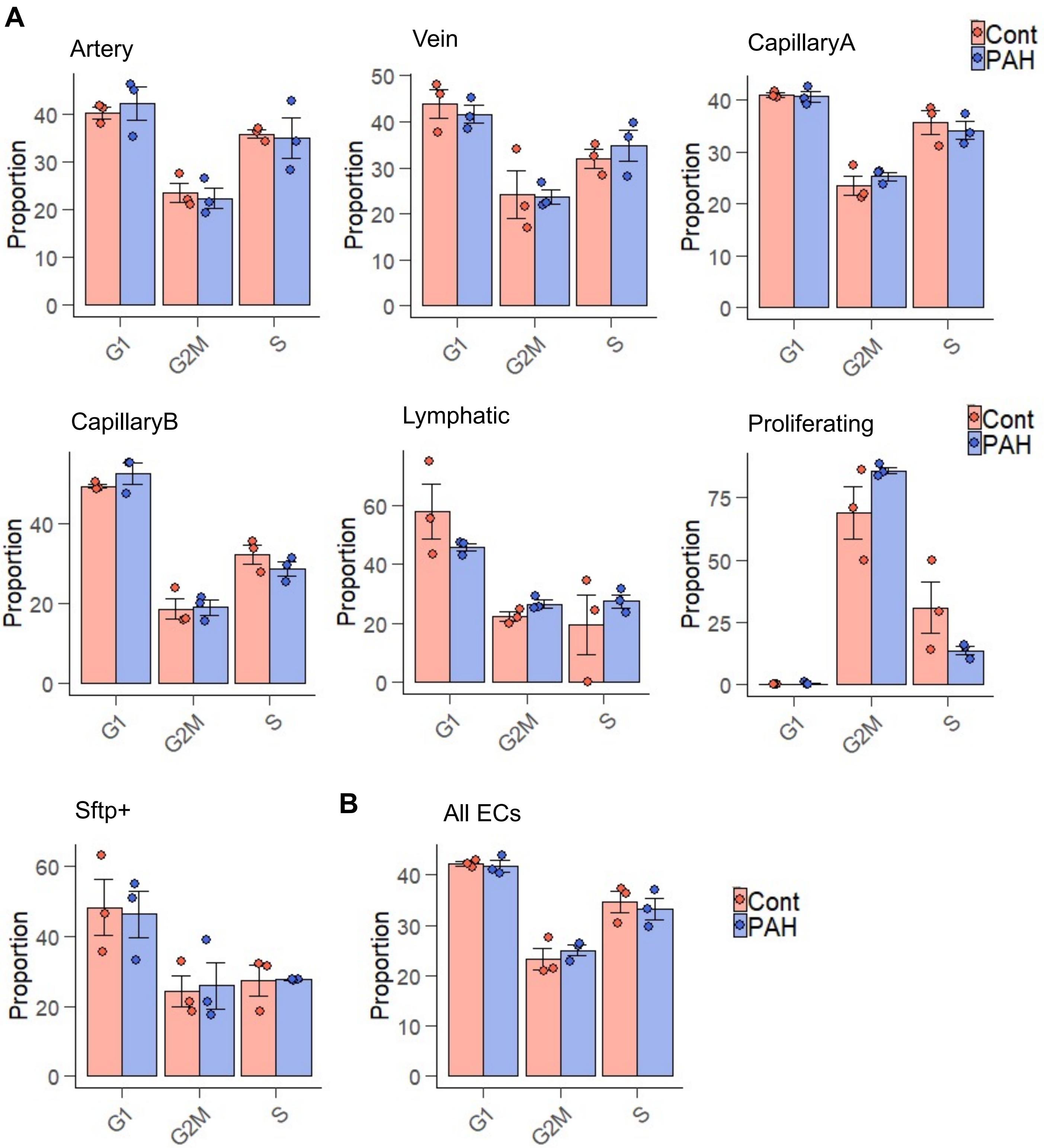

Supplementary Figure S10

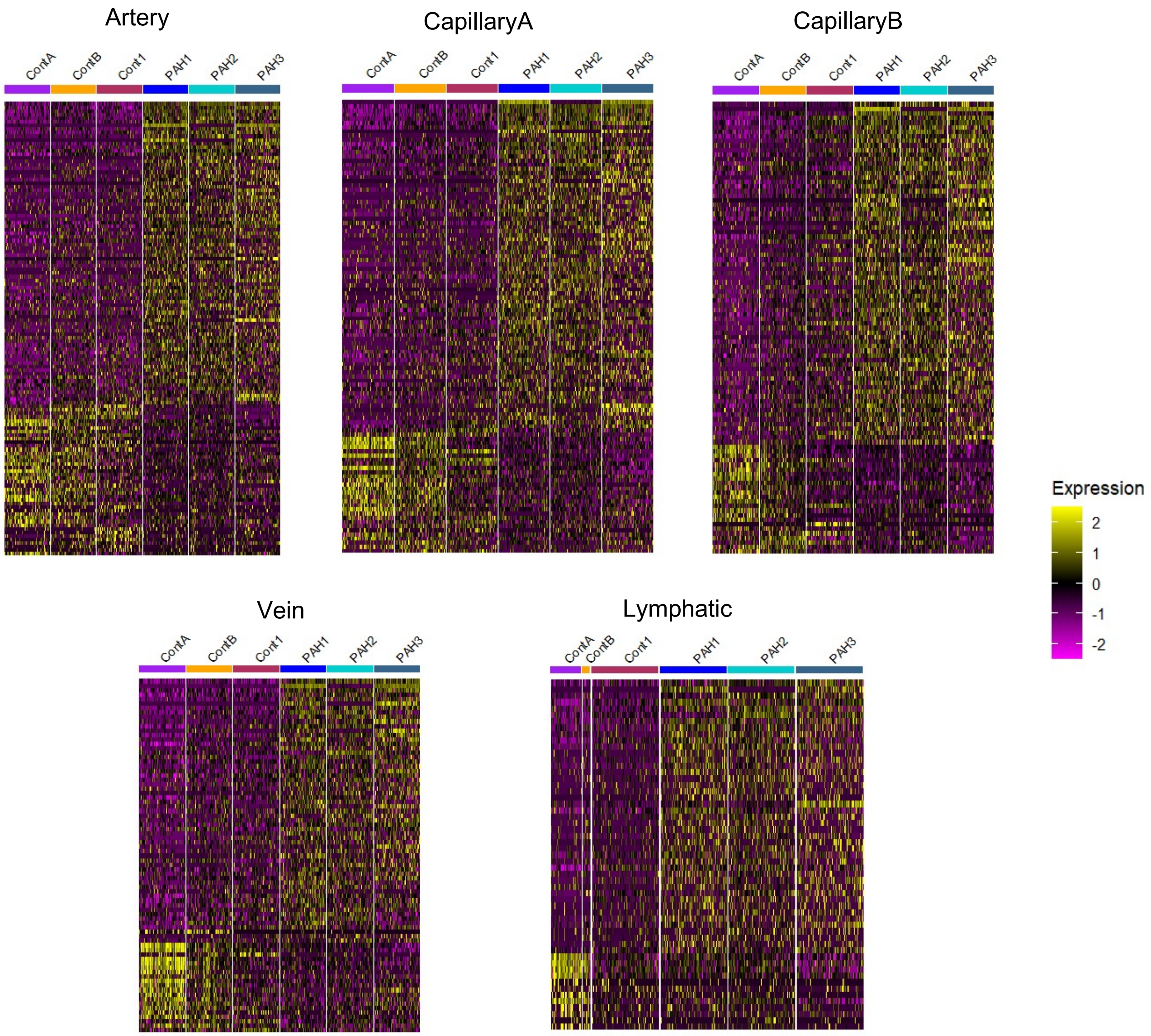

Supplementary Figure S11

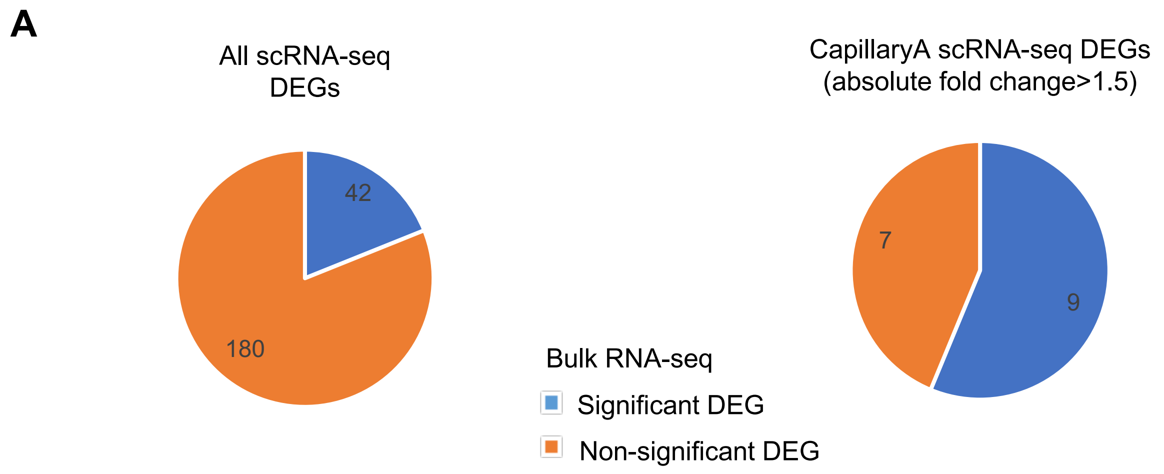

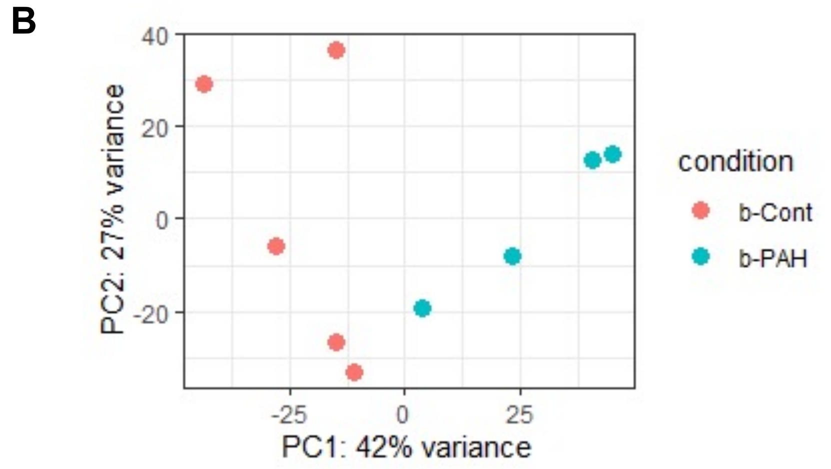

Supplementary Figure S12

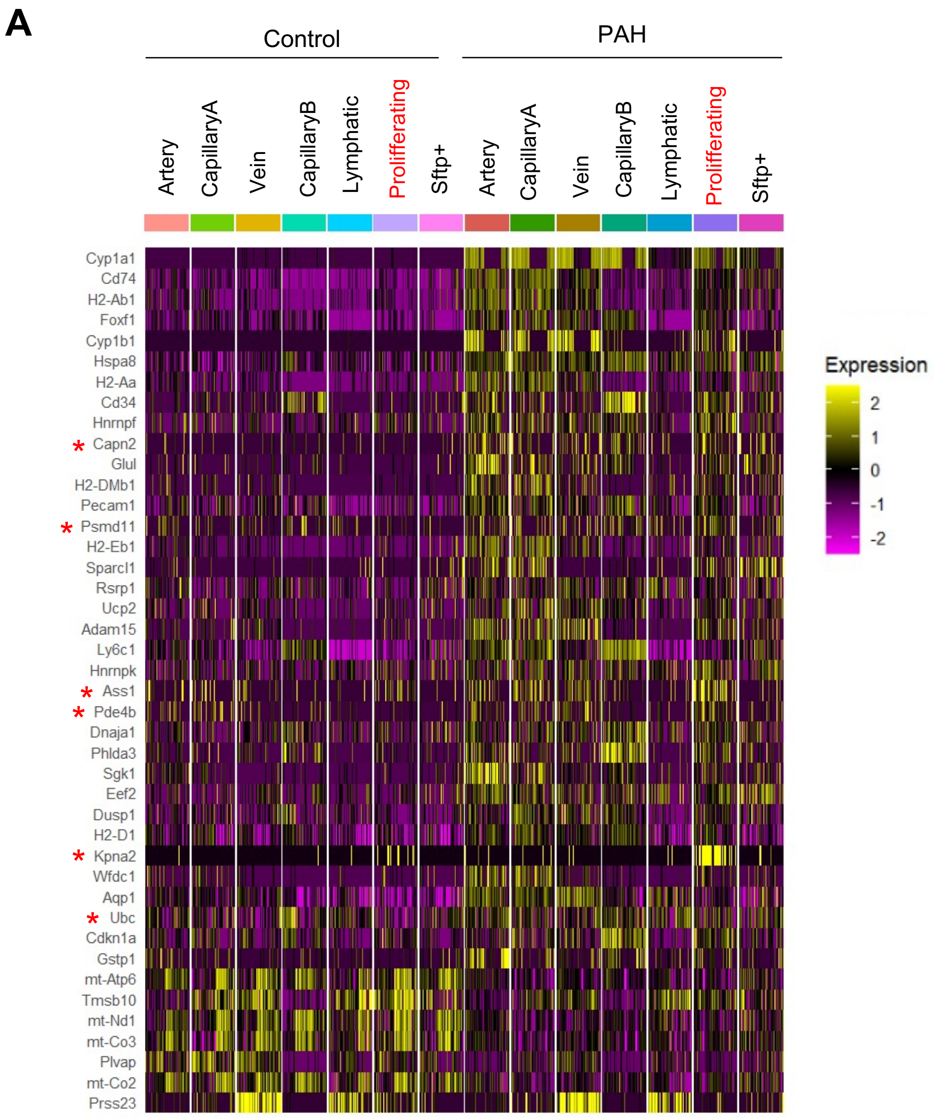

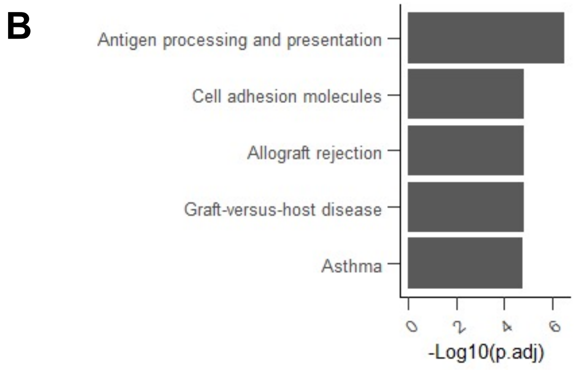

Supplementary Figure S13

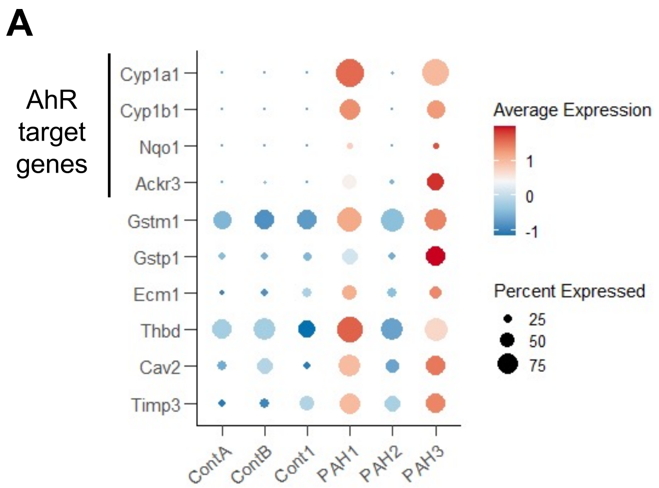

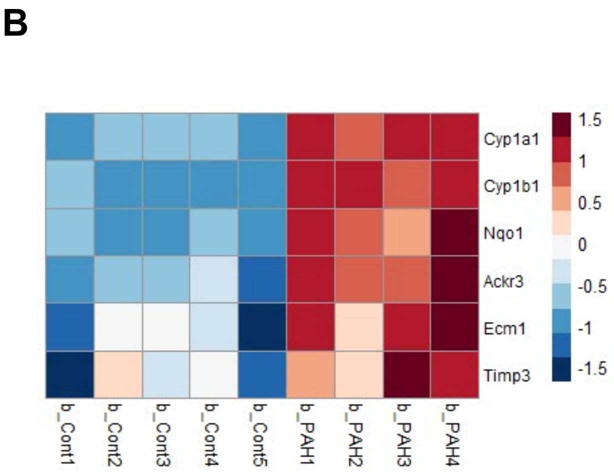

Supplementary Figure S14

A

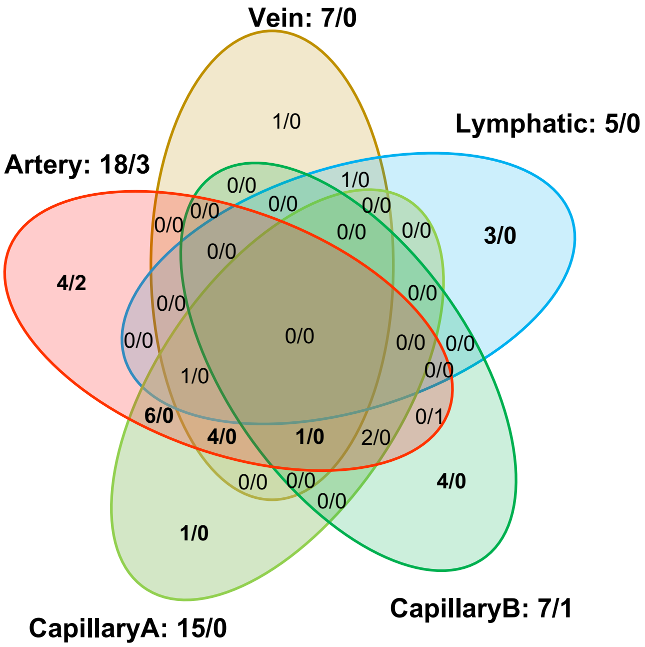

B

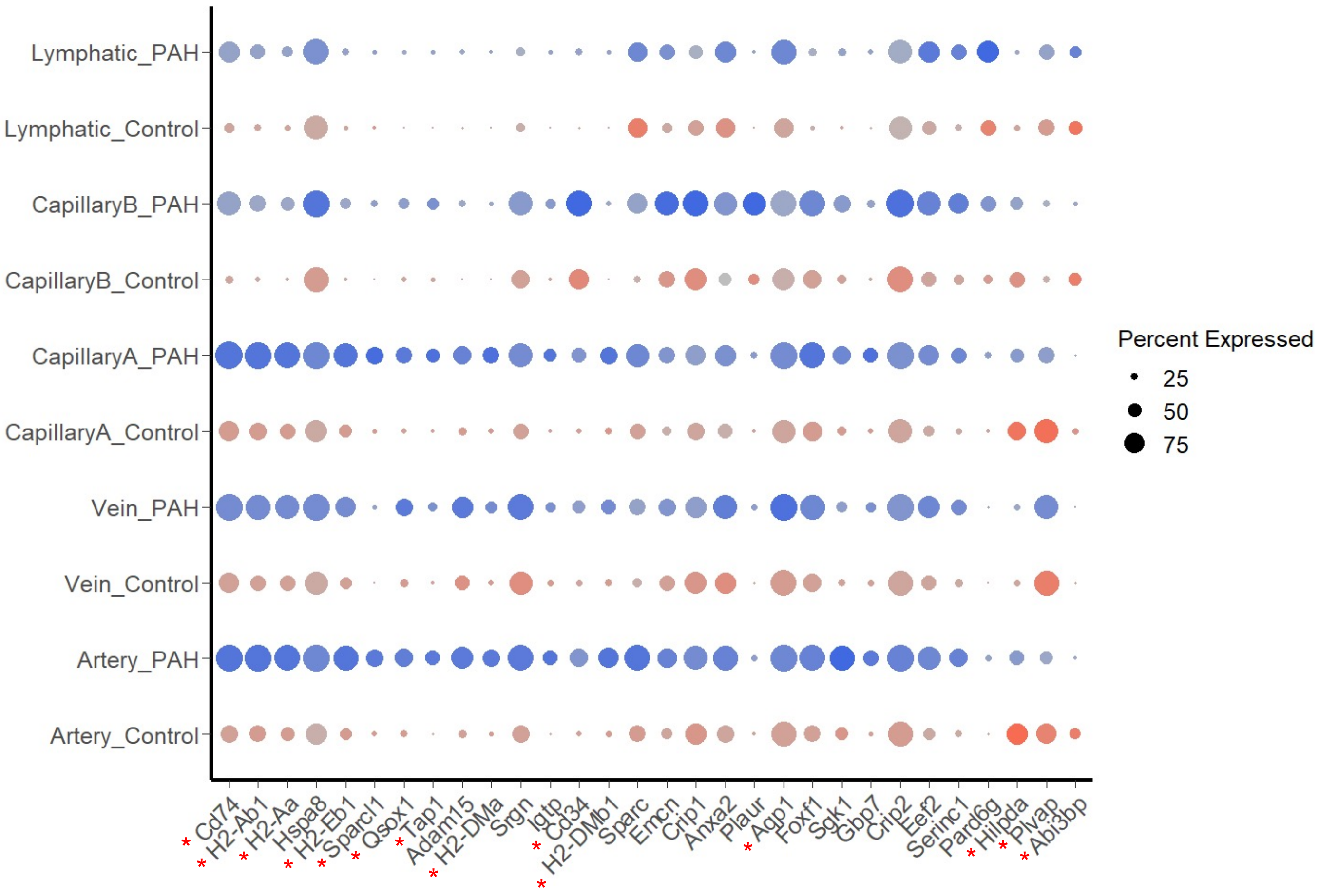

Supplementary Figure S15

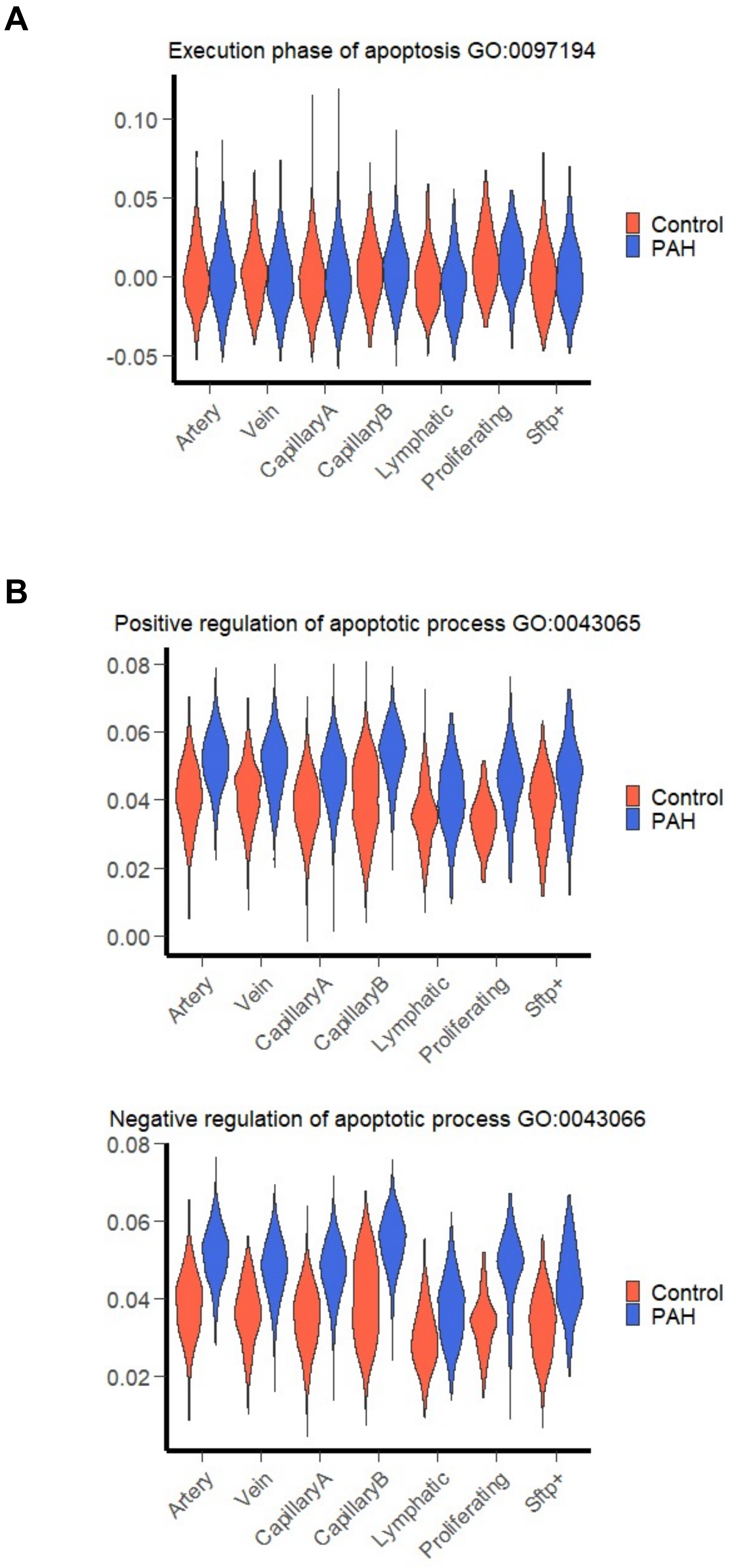

Supplementary Figure S16

A

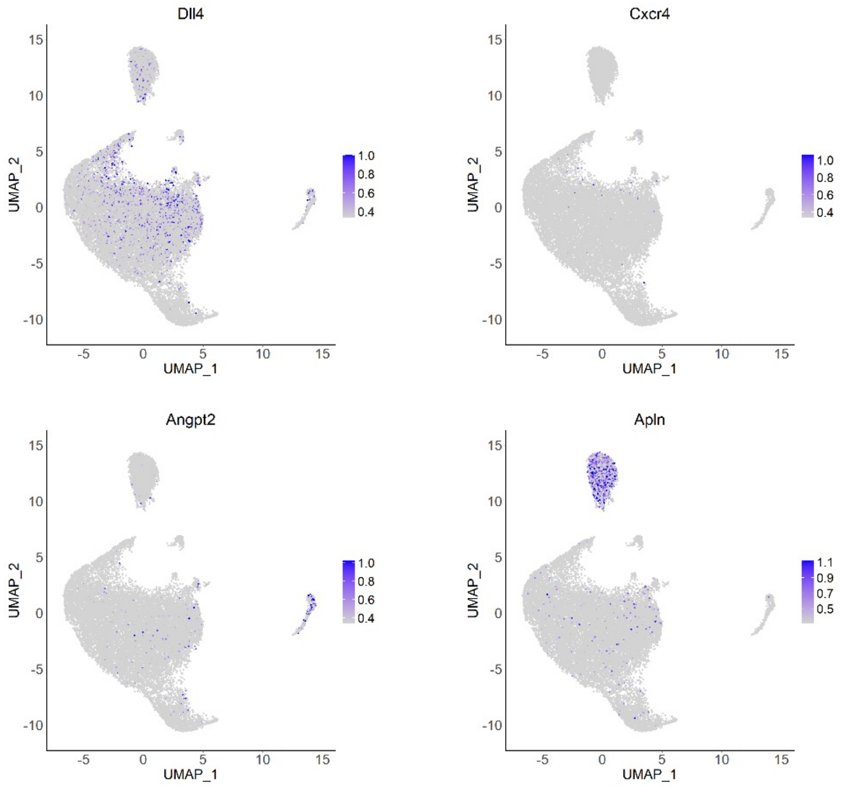

B

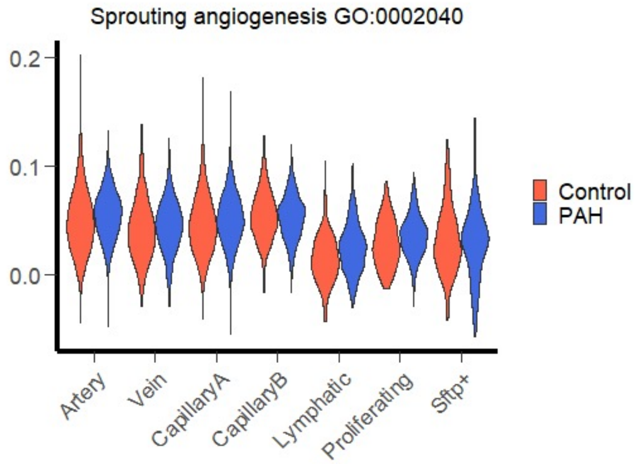

C

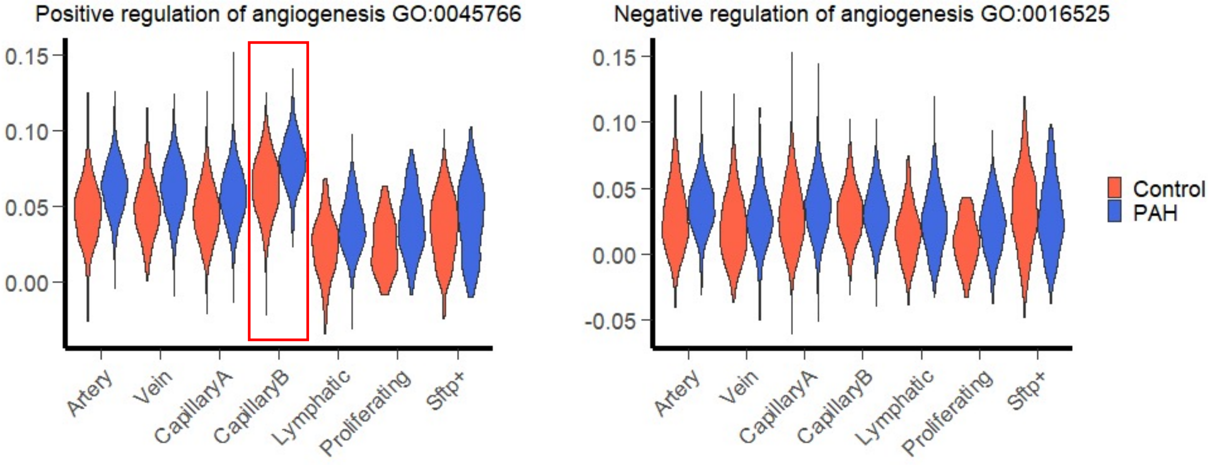

Supplementary Figure S17

A Rat scRNA-seq

	Total high quality cells	Endothelial cell number
Rat_Control1	999	9
Rat_Control2	1630	2
Rat_Control3	1079	14
Rat_Control4	5494	343
Rat_Control5	890	41
Rat_Control6	1721	49
Rat_MCT1	689	4
Rat_MCT2	838	3
Rat_MCT3	660	1
Rat_MCT4	2126	13
Rat_MCT5	3084	21
Rat_MCT6	2951	62
Rat_SuHx1	1328	4
Rat_SuHx2	1198	3
Rat_SuHx3	945	3
Rat_SuHx4	3147	24
Rat_SuHx5	2353	75
Rat_SuHx6	2260	87
Total	33392	758

B

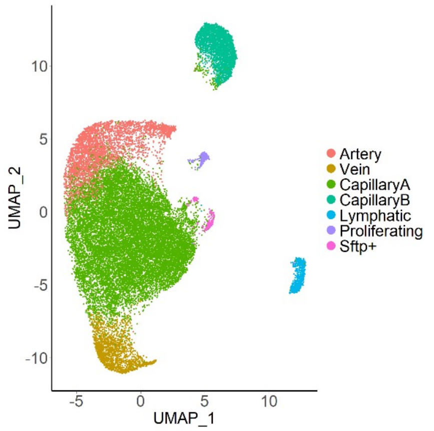

C

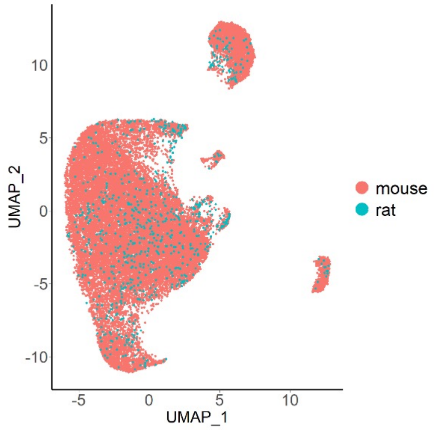

D

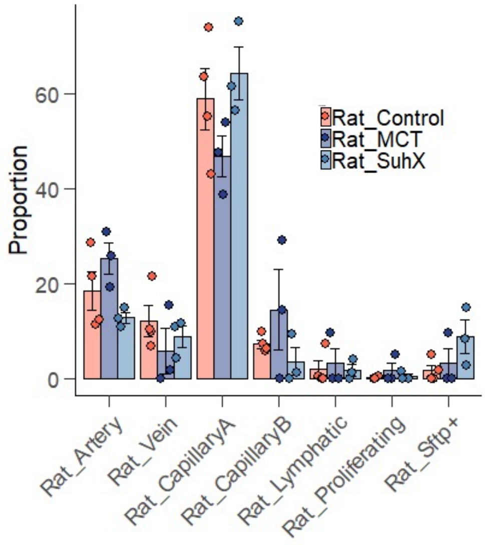

E

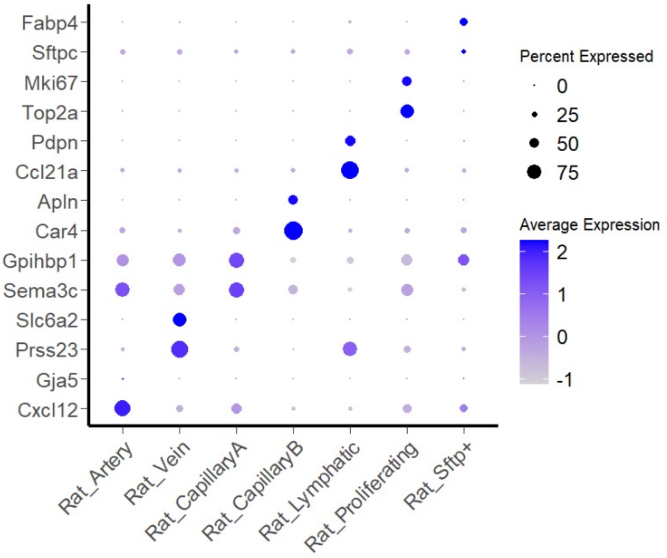

F

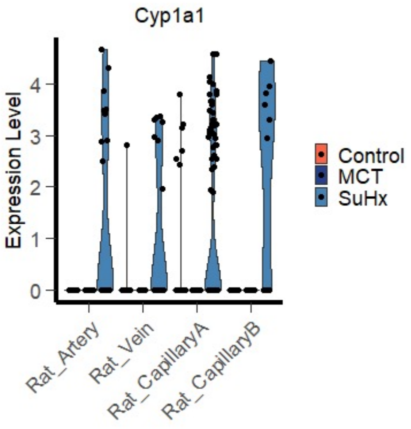

Supplementary Figure S18

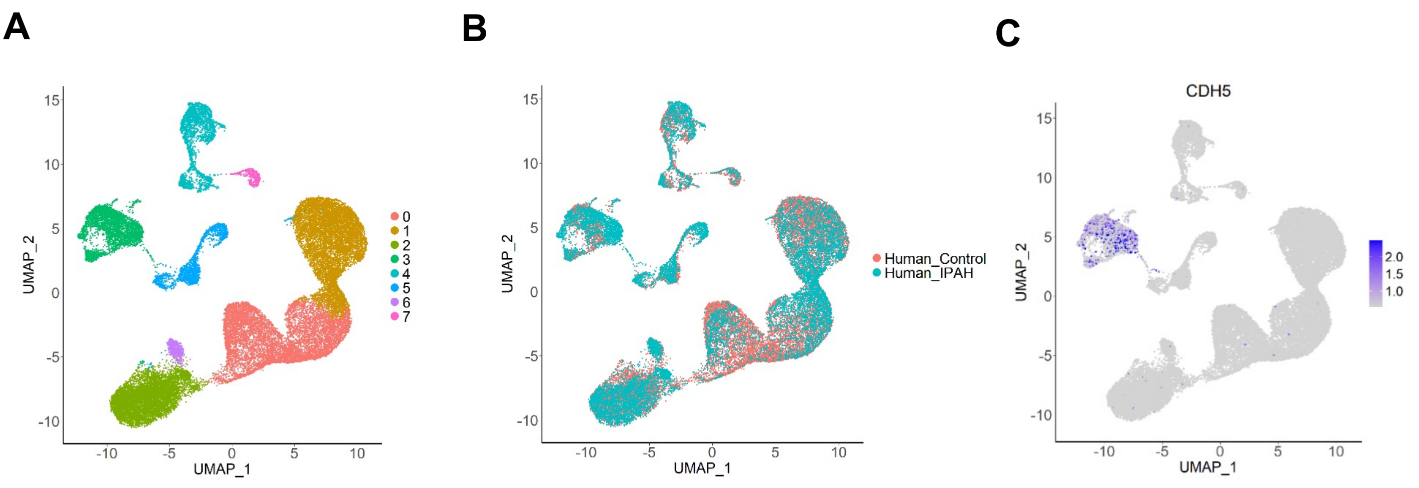

D

Human scRNA-seq

	Total high quality cells	Endothelial cell number
Human_Cont1	9399	978
Human_Cont2	2098	53
Human_Cont3	3122	45
Human_Cont4	4184	350
Human_Cont5	2933	269
Human_IPAH1	5185	461
Human_IPAH2	5045	657
Human_IPAH3	4358	1137
Total	36324	3950

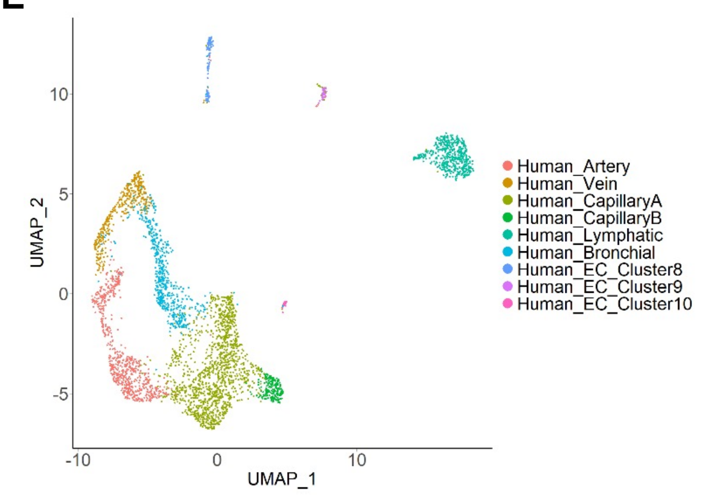

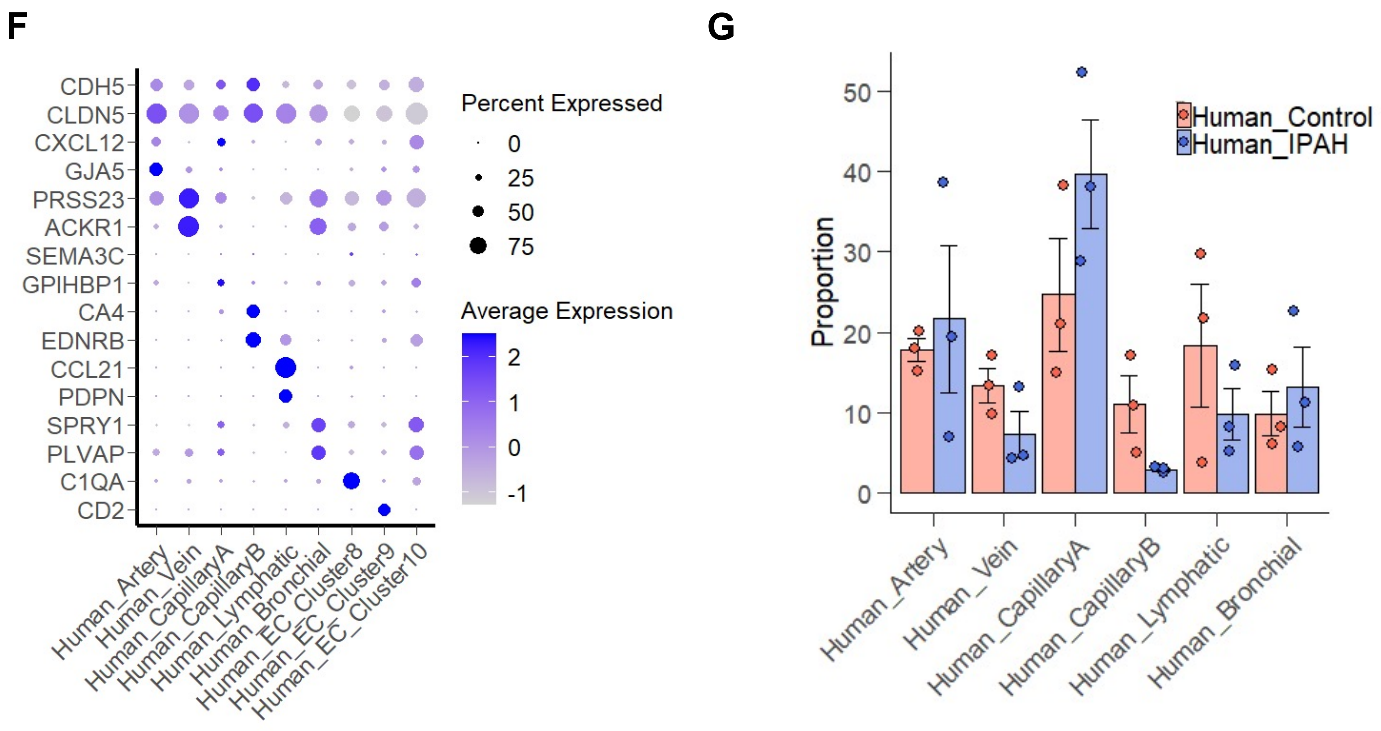

Supplementary Figure S19

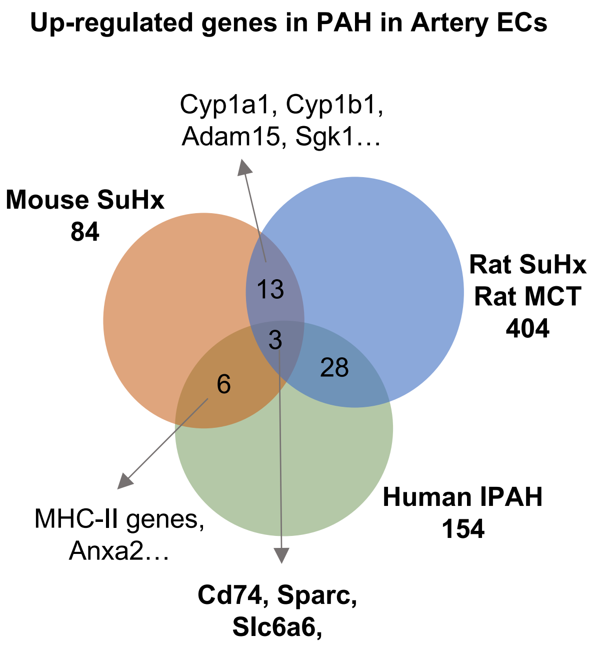

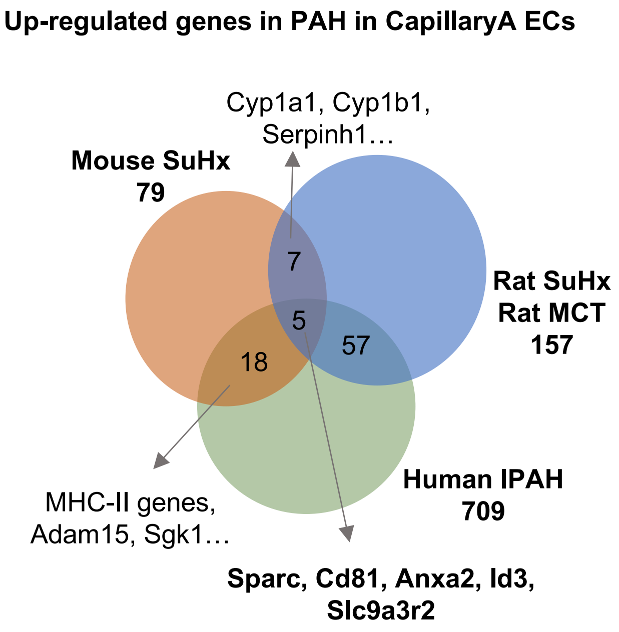

Supplementary Figure S20

A

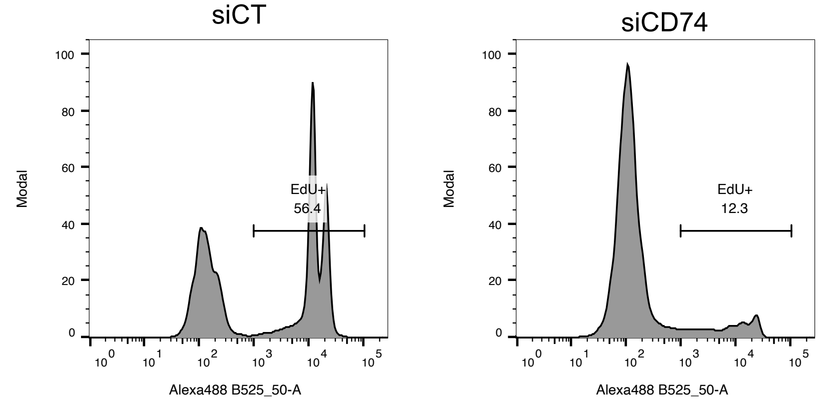

B

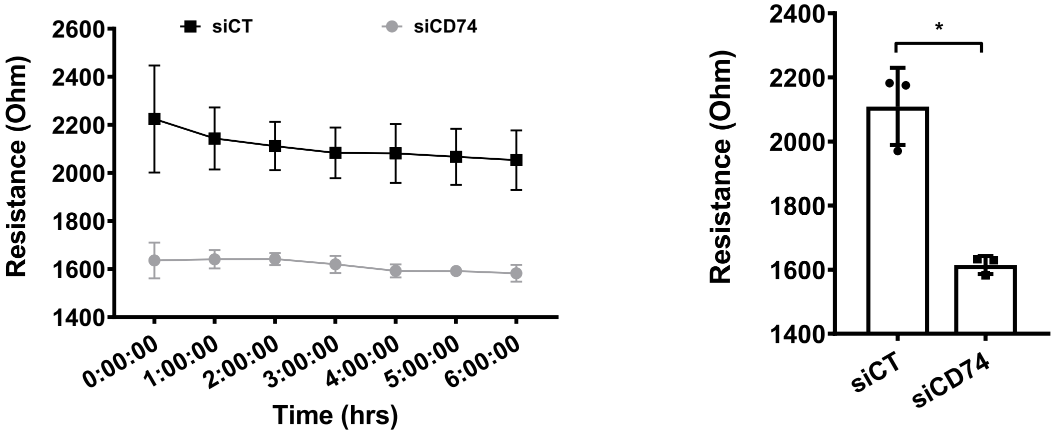

C

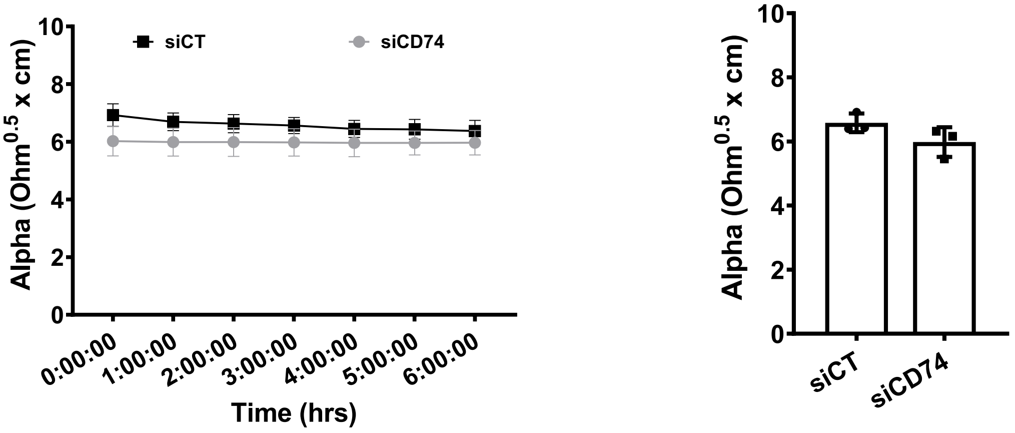

Supplementary Figure Legends

Supplementary Figure S1: Flow cytometry sorting strategy of mouse lung TdTomato+ cells.

A. Forward scatter (FSC) singlets gate, to
B. Side scatter (SSC) singlets gate, to
C. Gating for live cells, to
D. FSC/SSC selection for cells based on their size and granularity, to
E. Gating for TdTomato+ cells.

Supplementary Figure S2: Validation of the SuHx model in *Cdh5-CreERT2-TdTomato* and *C57BL/6* mouse.

A. Right ventricular systolic pressure (RVSP) and right ventricular hypertrophy (RV/LV+S) in *Cdh5-CreERT2-TdTomato* mice (n=6 Control and n=7 SuhX/PAH) and *C57/BL6* mice (n=5 Control and n=9 SuHx/PAH). The types of samples or downstream analysis of samples collected from each mouse is indicated. Statistical analysis was done using an unpaired t-test.
B. Percentage of remodelled arterioles in Control and SuHx *C57/BL6* mice determined by a-smooth muscle actin (SMA) staining. Statistical analysis of the changes of fully muscularised vessels (right panel) was done using a Mann-Whitney test.

Supplementary Figure S3: Single-cell RNA-seq of lung TdTomato+ cells from 2 Control mice after Tamoxifen injection.

A. Table summarising the quality control of the scRNA-seq data based on the output of CellRanger and Scater.
B. Uniform Manifold Approximation and Projection (UMAP) plot of merged data. Colours represent cell clusters, samples, and *TdTomato* expression, respectively.

Supplementary Figure S4: Table summarising the quality control of the scRNA-seq data from Experiment 2 based on the output of CellRanger and Scater.

Supplementary Figure S5: Cell type identification of the merged scRNA-seq data.

A. TdTomato expression (mean) and cell number per defined clusters.
B. Violin plot of *Pecam1* (pan EC marker) expression.
C. Violin plot of *Lgals3* (immune marker) and *Myl9* (fibroblast marker) expression.

Supplementary Figure S6: Limited contribution of endothelial to mesenchymal transition (EndMT) to the lung of Control and PAH mice.

A. UMAP plot of *Pecam1* expression (endothelial cell marker).
B. UMAP plot of *Acta2* and *Col1a1* expression (mesenchymal cell markers).
C. UMAP plot of *Snai1*, *Snai2*, *Zeb2* and *Smad3* expression (EndMT regulators).
D. Proportion of Acta2+ cells from the TdTomato+ cells in Control and PAH samples. Error bars represent the standard error around the mean.
E. Proportion of Col1a1+ cells from the TdTomato+ cells in Control and PAH samples. Error bars represent the standard error around the mean.
F. Expression level of *Acta2*, *Col1a1*, *TdTomato* and *Cdh5* in the Acta2- and Acta2+ TdTomato+ population.

Supplementary Figure S7: Identification of cell subpopulations in merged Control samples.

A. UMAP plot of the merged Control samples. Colours represent identified clusters and individual sample respectively.
B. Proportion of each cluster in Control samples.

Supplementary Figure S8: Identification of endothelial cell subpopulations in the integrated PAH samples.

A. UMAP plot of integrated PAH samples. Colours represent the 7 annotated clusters and individual sample, respectively.
B. Proportion of annotated EC subpopulations in individual PAH samples.
C. Heatmap showing the expression of the top 10 marker genes in a downsampling of 100 cells from each EC subpopulation.
D. UMAP plot of representative marker expression in EC subpopulations.

Supplementary Figure S9: Proportion of cells in each cell cycle phase between Control and PAH in EC subtypes (A) and all ECs (B) based on Seurat cell cycle scoring.

Supplementary Figure S10: Expression profile of the differential expressed genes in the Control and PAH replicates. Heatmap of the differential expressed genes obtained in each population across the Control and PAH samples.

Supplementary Figure S11: Validation of the DEG scRNA-seq by bulk RNA-seq.

A. Number of scRNA-seq DEGs with validated significant change based on bulk RNA-seq.
B. Principal component analysis of the bulk RNA-seq.

Supplementary Figure S12: PAH Response of Proliferating ECs.

A. Heatmap of the 42 differentially expressed genes (DEGs) between PAH and Control in Proliferating ECs across EC populations and conditions. Changes not observed in the vessel type EC subtypes are indicated by a red star.
B. Enriched KEGG pathway for the 42 DEGs between PAH and Control in Proliferating ECs.

Supplementary Figure S13: Expression of DEG with inter-individual variability.

A. Dot plot showing the expression of 10 genes up-regulated in PAH1 and PAH3 samples compared with PAH2. The four target genes of the transcription factor AhR activated by Sugen-5416 are indicated.
B. Heatmap based on the bulk RNA-seq (z-score of Log2(FPKM+1)) of genes without any up-regulation in PAH2 (scRNA-seq) but significant up-regulation in the bulk RNA-seq.

Supplementary Figure S14: Identification of a stringent differential expressed gene set.

A. Venn diagram of differential gene expression changes identified in 5 vessel type EC subpopulations with a stringent approach (individual analysis of each PAH versus each Control samples). Number of changes are indicated as follow: Number of up-regulated genes/Number of down-regulated genes.
B. Dot plot of the stringent differential expressed gene expression set across EC populations and conditions. The red star indicates a significant regulation in the bulk RNA-seq.

Supplementary Figure S15: Regulation of apoptosis pathway in EC subpopulations in Control and PAH samples.

Signature score of genes involved in **(A)** execution phase of apoptosis and **(B)** positive/negative regulation of apoptotic process across EC types and conditions.

Supplementary Figure S16: Tip cell and angiogenesis pathway analysis in Control and PAH samples.

A. UMAP plot of 4 tip-cell marker expression: *Dll4*, *Cxcr4*, *Angpt2*, *Apln*.
B. Signature score of genes involved in sprouting angiogenesis across EC types and conditions.

C. Signature score of genes involved in positive/negative regulation of angiogenesis across EC types and conditions.

Supplementary Figure S17: Endothelial cell subtypes in rat lung scRNA-seq from Control, SuHx –induced and MCT-induced samples (dataset from Hong *et al*., 2021).

A. Table of total and endothelial cell numbers in the different Control, SuHx and MCT samples.
B. UMAP plot of EC type annotation of the rat EC dataset integrated to the mouse EC dataset.
C. UMAP plot showing species contribution on the rat and mouse integrated data.
D. Relative proportion of rat EC subtypes across Control and PAH models (MCT and SuHx).
E. Dot plot of EC type markers in the annotated rat EC subtypes.
F. *Cyp1a1* expression across rat EC subtypes and conditions.

Supplementary Figure S18: Identification of endothelial population in scRNA-seq from Control and IPAH human lungs (raw data obtained from Saygin *et al*., 2020).

UMAP plot of (A) clusters (B) condition (C) CDH5 expression.

D. Table of total cell and ECs number across samples.
E. UMAP plot of ECs after integration of ECs from all samples with EC population annotation.
F. Dot plot of EC markers across EC populations.
G. Relative proportion of all EC subtypes across conditions (Cont2 and Cont3 not included due to their low number of ECs).

Supplementary Figure S19: Overlap of up-regulated genes in mouse, rat and human Artery (left panel) and CapillaryA (right panel) EC populations in PAH.

Supplementary Figure S20: CD74 regulates HUVEC proliferation and barrier function

A. Representative graph of EdU incorporation in Control (siCT) and CD74 (siCD) knockdown HUVEC.
B. Barrier resistance (Ohm) in siCT and siCD74 HUVEC across a 6h time course with bar graph showing the average across the time points (n=3).
C. Cell-matrix interaction ($Ohm^{0.5}$xcm) in siCT and siCD74 HUVEC across a 6h time course with bar graph showing the average across the time points (n=3).

Graph in panel B and C correspond to mean ± standard error of the mean and p-values were obtained using an unpaired t-test. * p-value<0.05.
